# Comparative performance of EQ-5D-5L bolt-ons in China and the Netherlands: results from the EQ-DAPHNIE project

**DOI:** 10.1007/s11136-026-04328-3

**Published:** 2026-06-28

**Authors:** Seo-Ho Cho, Mathieu F. Janssen, Fatima Al Sayah, Zhuxin Mao, You-Shan Feng, Lajos V. Kemény, Fanni Rencz

**Affiliations:** 1 General Medicine, Semmelweis University, Budapest, Hungary; 2 Károly Rácz Conservative Medicine Division, Doctoral College, Semmelweis University, Budapest, Hungary; 3 Department of Dermatology, Venereology and Dermatooncology, Semmelweis University, Budapest, Hungary; 4 Department of Physiology, Semmelweis University, Budapest, Hungary; 5 HCEMM-SU Translational Dermatology Research Group, Semmelweis University, Budapest, Hungary; 6 Section Medical Psychology and Psychotherapy, Department of Psychiatry, Erasmus MC, Rotterdam, The Netherlands; 7 Alberta PROMs and EQ-5D Research and Support Unit (APERSU), School of Public Health, University of Alberta, Edmonton, AB Canada; 8 Centre for Health Economics Research and Modelling Infectious Diseases (CHERMID), University of Antwerp, Antwerpen, Belgium; 9 Institute for Clinical Epidemiology and Applied Biostatistics, Universitätsklinikum Tübingen, Tübingen, Germany; 10 MTA-SE Lendület “Momentum”, Dermatology Research Group, Hungarian Academy of Sciences and Semmelweis University, Budapest, Hungary; 11 Institute of Social and Political Sciences, Corvinus University of Budapest, Budapest, Hungary; 12 EuroQol Research Foundation, Rotterdam, The Netherlands

**Keywords:** EQ-5D-5L, Bolt-on, EQ-5D Bolt-on Toolbox, Health-related quality of life, Psychometrics, Cross-cultural comparison

## Abstract

**Purpose:**

This study compares the psychometric properties of eight EQ-5D-5L bolt-ons in the general populations from two culturally distinct countries: China (CN) and the Netherlands (NL).

**Methods:**

As part of the EQ-DAPHNIE project, cross-sectional online surveys were conducted in 2024 with quota-based samples approximating the adult population from CN and the NL. Participants completed EQ-5D-5L, eight bolt-ons (vision, hearing, breathing, sleep, tiredness, social relationships, self-confidence, and cognition/memory), and sociodemographic and health-related questions. Psychometric assessments included ceiling, convergent/divergent validity, structural validity, known-groups validity, and explanatory power.

**Results:**

The ceiling of the EQ-5D-5L was higher in CN (45%) than NL (32%). In both countries, vision, tiredness, and sleep bolt-ons reduced the ceiling the most, while breathing had the least effect. Tiredness and self-confidence bolt-ons demonstrated stronger associations with the core dimensions. Several bolt-ons significantly improved known-groups validity. In CN, hearing showed stronger known-groups discrimination across age groups, while self-confidence had the greatest impact in NL. The core dimensions explained more variance in EQ VAS in NL (37%) than CN (25%). In both countries, tiredness and self-confidence contributed most to increasing the variance explained. Principal component analysis (PCA) confirmed a seven-factor structure, with all bolt-ons loading separately from the core dimensions. In country-specific PCAs, no major differences were observed between CN and NL.

**Conclusion:**

While certain bolt-ons demonstrated robust psychometric properties in both countries, others showed greater context dependence. These findings add to the growing body of evidence supporting the development of the experimental EQ-5D Bolt-on Toolbox.

**Supplementary Information:**

The online version contains supplementary material available at https://doi.org/10.1007/s11136-026-04328-3.

## Abstract (highlights)

Measuring how health problems affect people’s daily lives is important for informing healthcare decisions. One widely used tool around the world is the EQ-5D-5L, but it may not cover some important aspects of people’s health. This study explored whether adding extra questions, called bolt-ons, about areas like tiredness, sleep, vision, and social relationships could make the tool more comprehensive and informative. Over 9000 adults from China and the Netherlands took part, answering the original EQ-5D-5L questions, eight bolt-ons, and additional questions about their health and background. The study found that certain bolt-ons, such as tiredness, sleep, and vision, captured health aspects that the original tool may have missed. The effectiveness of these extra questions varied between the two countries, suggesting that cultural contexts can affect how people experience and report their health. Overall, these findings suggest that carefully selected bolt-ons may provide additional information on health dimensions that are not fully captured by the EQ-5D-5L across population subgroups.

**Supplementary Information:**

The online version contains supplementary material available at https://doi.org/10.1007/s11136-026-04328-3.

## Introduction

The EQ-5D-5L is a widely used generic preference-accompanied health-related quality of life (HRQoL) instrument with a descriptive system consisting of five dimensions: mobility, self-care, usual activities, pain/discomfort, and anxiety/depression [1]. Although it has demonstrated robustness across a wide range of populations, the EQ-5D-5L may not be able to capture the full spectrum of HRQoL, particularly in certain clinical conditions or population groups [2]. One notable limitation is its relatively high ceiling in some populations, which can reduce sensitivity in detecting milder HRQoL impairments [3]. To address these gaps, additional dimensions targeting specific HRQoL dimensions, referred to as “bolt-ons”, were proposed. These bolt-ons are supplementary dimensions designed to enhance the comprehensiveness of EQ-5D-5L by capturing HRQoL aspects not covered by its core dimensions, such as sensory problems, vitality/fatigue, sleep, or cognitive functioning [4–6].

While several bolt-ons have been explored in specific clinical contexts [7–17], there remains a lack of large-scale multi-country studies that have comprehensively assessed their psychometric performance across various population groups. It is important to understand the cross-cultural validity of these bolt-ons, as health perceptions and reporting behaviors are often shaped by cultural values and societal norms. Notably, cultural differences between Eastern and Western populations may influence how individuals interpret and respond to HRQoL instruments. Studies have shown that Chinese views on health, incorporating elements like food and weather, differ from Western perspectives [18, 19]. Furthermore, a systematic review examining the ceiling of EQ-5D-5L across 37 countries found that East/Southeast Asia exhibited, on average, 9% higher ceiling compared to Europe, suggesting that cultural contexts may significantly impact the way health status is perceived and reported [3].

These cultural distinctions raise important questions about the validity and comparability of HRQoL measures when applied in different settings. Bolt-ons have been increasingly used in multi-country population and patient surveys, population norm studies and clinical trials [20–24]. With the experimental EQ-5D Bolt-on Toolbox™ becoming available, the use of bolt-ons is expected to further increase [25]. However, their cross-cultural validity and comparability are less known. This study focuses on eight bolt-ons (breathing, cognition/memory, vision, hearing, sleep, tiredness, self-confidence, and social relationships) that have been implemented within the EuroQol Data for Assessment of Population Health Needs and Instrument Evaluation (EQ-DAPHNIE) project and administered in general population surveys, noting that these bolt-ons may differ slightly in wording from the experimental EQ-5D Bolt-on Toolbox [26]. Although the EQ-DAPHNIE project includes multiple countries, the full set of these eight bolt-ons was included only in China and the Netherlands due to the availability of translations in these countries. Accordingly, this study aims to compare the psychometric performance of the abovementioned eight bolt-ons in the general populations of two culturally distinct countries, China (CN) and the Netherlands (NL).

## Methods

### Study design and recruitment

A cross-sectional online survey using online panels was conducted in 2024 among the adult general populations of China and the Netherlands as part of the EQ-DAPHNIE project [26]. Eligible participants were adults aged 18 years or older who were able and willing to complete the survey in their country’s official language and who provided informed consent prior to participation. A quota sampling approach was employed to achieve a representative sample in each country. Each country had a target sample size of 4,500 respondents, with quotas based on key demographic variables, including age, sex, household income, and area of residence. All participants completed a standardized core questionnaire that included the EQ-5D-5L, bolt-ons, and other validated external instruments. Additional information was collected on the participants’ sociodemographic characteristics and self-reported chronic health conditions. Participants were able to omit any questions in the survey. Further details regarding the study protocol, design, recruitment procedures, and survey implementation can be found elsewhere [26, 27]. As this study involved a secondary analysis of previously collected survey data, patients or members of the public were not involved in the design, conduct, reporting, or dissemination of this study.

### Outcome measures

#### EQ-5D-5L and bolt-ons

The EQ-5D-5L is a standardized instrument for measuring HRQoL, consisting of two components: a descriptive system and a visual analogue scale (EQ VAS) [1]. The descriptive system comprises five dimensions, with each dimension rated on a five-level scale ranging from “no problems” (level 1) to “extreme problems/unable to” (level 5), with a recall period of “today”. The EQ VAS records the respondent’s overall self-rated health on the day of completion, using a vertical thermometer ranging from 0 (“the worst health you can imagine”) to 100 (“the best health you can imagine”).

This study also included eight previously developed single-item EQ-5D-5L bolt-ons that were asked in the fixed order provided by the EQ-DAPHNIE dataset: vision, hearing, breathing, sleep, tiredness, social relationships, self-confidence, and cognition/memory, following the five core dimensions and the EQ VAS [26]. All these dimensions, with the exception of social relationships, have been proposed for inclusion in the experimental EQ-5D Bolt-on Toolbox, a curated collection of bolt-ons intended for use alongside the EQ-5D-5L in well-defined circumstances [28]. However, with the exception of self-confidence, the wording of the bolt-ons in EQ-DAPHNIE (Appendix 1) differed from that used in the experimental EQ-5D Bolt-on Toolbox (version no. EV1.0). The bolt-ons were worded identically across the two countries, except for the cognition/memory bolt-on, which was labeled “cognition (memory, comprehension, concentration, thinking)” in the Dutch survey and “memory” in the Chinese survey. These items therefore do not represent fully equivalent constructs, with the Dutch version assessing a broader cognitive domain and the Chinese version focusing specifically on memory. Since this is a psychometric study and given that value sets for the bolt-ons are not yet available, we did not calculate an index value for the core dimensions either. Additional outcome measures used in the study are described in Appendix 2.

### Statistical analysis

#### Distributional characteristics

Descriptive analyses were conducted to compare demographic and health-related characteristics of participants from China and the Netherlands. Chi-square tests and Fisher’s exact tests were used, as appropriate, to assess differences between the two country samples. Responses indicating an age over 100 years were treated as data entry errors and coded as missing. Subsequently, we examined the distribution of responses across all EQ-5D-5L and bolt-on dimensions for each country sample to explore the potential differences in reporting patterns. Ceiling was assessed by calculating the proportion of participants reporting the best possible response for each item and for all items of the instrument (i.e. EQ-5D-5L + (bolt-on(s)). To assess the incremental information provided by each bolt-on or combination of bolt-ons, we calculated both the absolute and relative reduction in ceiling generated by their addition. Bolt-ons were added sequentially based on their individual ability to reduce ceiling when added to the EQ-5D-5L. Specifically, bolt-ons associated with the greatest reduction in ceiling were added first, followed by those with progressively smaller effects. This approach was used to illustrate the cumulative impact of adding bolt-ons with the highest potential to reduce ceiling, rather than to imply a recommended order of bolt-on use in practice.

Propensity score matching was conducted as a sensitivity analysis to reduce potential confounding due to sociodemographic differences between China and the Netherlands. Respondents were matched on age and sex. Following matching, the distribution of bolt-on responses and ceiling was examined across countries.

#### Convergent and divergent validity

Convergent and divergent validity was evaluated using the Spearman’s rank-order correlations between the bolt-ons and the core dimensions as well as conceptually related constructs from other validated instruments (PROMIS Global Health [29], PROMIS Sleep Disturbance Short Form [30], ICECAP-A [31], WHO-5 well-being index [32], and OPQOL-brief (the latter only displayed to respondents aged 65 + years) [33]). Correlation strength was interpreted as very weak (< 0.20), weak (0.20–0.39), moderate (0.40–0.59), or strong (≥ 0.60) [34]. Hypotheses were specified *a priori* based on the degree of conceptual and functional overlap between each bolt-on and the EQ-5D-5L dimensions. Bolt-ons were categorized into five broad constructs: symptom burden (sleep and tiredness), physical functioning (breathing), executive functioning (cognition/memory), psychosocial functioning (self-confidence and social relationships), and sensory functioning (vision and hearing). Moderate correlations were expected when a bolt-on and EQ-5D-5L dimensions reflected related underlying constructs, whereas weak correlations were expected for conceptually distinct constructs. Therefore, moderate correlations were hypothesized between tiredness and sleep with usual activities, pain/discomfort, and anxiety/depression; breathing with mobility, self-care, and usual activities; cognition/memory with anxiety/depression; and self-confidence and social relationships with anxiety/depression [35–37]. In contrast, sensory functioning bolt-ons (vision and hearing) were expected to show weak correlations with the EQ-5D-5L dimensions, because they assess more specific functional capacities that may not directly translate into limitations in broader health domains, particularly in the presence of compensatory strategies or assistive devices. All remaining bolt-on and EQ-5D-5L dimension pairs were expected to show weak or very weak correlations. For convergent validity with external instruments, we hypothesized at least moderate correlations between bolt-ons and conceptually aligned items in these instruments.

#### Structural validity

Structural validity of EQ-5D-5L and the eight bolt-ons were examined using principal component analysis (PCA). The primary aim of this analysis was to assess whether the bolt-ons capture constructs that are distinct from the core dimensions, thereby providing evidence on their added content validity. To support the identification and interpretation of latent constructs, items from additional validated instruments were included in the PCA. A total of 42 items from the EQ-5D-5L, bolt-ons, PROMIS Global Health, PROMIS Sleep Disturbance Short Form, ICECAP-A, WHO-5 well-being index, PHQ-2 [38], GAD-2 [39], and social safety net questionnaire instruments [40] were included. PCA was performed on the pooled dataset from both countries using promax rotation to allow for correlated components. Factor retention was primarily guided by Kaiser’s criterion (eigenvalue > 1), and was further supported by visual inspection of scree plots and parallel analysis [41]. These complementary criteria were used to assess the robustness of the retained factor solution and to reduce the risk of over- or under-extraction of components. Factor loadings were standardized and interpreted using the following benchmarks: ≤ 0.29 (unacceptable), 0.30–0.44 (poor), 0.45–0.54 (fair), 0.55–0.62 (good), 0.63–0.70 (very good), and ≥ 0.71 (excellent) [42]. Sensitivity analyses were conducted separately for each country dataset. Items with standardized factor loadings ≥|0.30| were reported and used to assign items to underlying latent constructs.

#### Known-groups validity

Known-groups validity was assessed by comparing mean level sum scores (LSSs) of the core dimensions alone and with combinations of bolt-ons across prespecified groups. All scores were transformed to a 0–100 scale for comparability, with lower scores indicating better HRQoL. Known groups were defined by self-reported chronic health conditions, age strata, and general, physical and mental health and quality of life (based on the first four items of PROMIS Global Health). For the chronic condition analyses, each condition group was compared to a reference group of participants who reported having no chronic conditions. Only chronic condition subgroups with at least 30 participants were included in the analysis. Furthermore, we only reported conditions where at least one bolt-on led to a statistically significant improvement in the discriminative performance of EQ-5D-5L. The selection of relevant bolt-ons for each chronic condition was informed by a review of the literature and expert judgement from the study team, which included two medical doctors. This selection was hypothesis-driven rather than data-driven, reflecting expected conceptual links between specific chronic conditions and HRQoL dimensions. The chronic condition-specific bolt-on combinations examined in the known-groups analyses are provided in Appendix 3. For analyses based on age strata and PROMIS Global Health groups, all bolt-ons were considered in both countries.

Differences in LSSs across groups were analyzed using ANOVA. Although candidate bolt-ons were identified *a priori* based on pre-specified hypotheses and conceptual relevance, subsequent analyses used statistical retention criteria to identify bolt-ons providing independent explanatory value within each country-specific model. The relative efficiency (RE) of detecting the differences between the known groups was calculated as the ratio of the F-statistic to that of the core dimensions alone. The RE value > 1 indicated that the addition of bolt-ons improved discriminative power. To test the statistical significance of RE improvements, 95% confidence intervals were estimated using 3,000 bootstrap replications. Within each country, bolt-ons were sequentially added to EQ-5D-5L in order of descending RE gain until no further statistically significant gain in RE was observed. Only the bolt-ons that showed statistically significant RE improvements were reported. Consequently, the bolt-on combinations reported for each known group may differ across countries, reflecting country-specific statistical performance. We hypothesized that relevant bolt-ons would improve the ability of EQ-5D-5L to distinguish between known groups.

As a sensitivity analysis, known-groups validity was additionally assessed while adjusting for potential confounding by demographic characteristics. Linear regression models were estimated to compare LSSs between respondents with and without chronic conditions while adjusting for age and sex. Adjusted estimated means with 95% confidence intervals were obtained from these models, and standardized effect sizes (Cohen’s d) were derived based on the adjusted estimates. Bolt-ons were sequentially added to the core dimensions in order of descending effect size until no further improvement in effect size was observed.

#### Explanatory power

Linear regression models were used to assess the explanatory power of bolt-ons in predicting HRQoL and well-being outcomes. Two outcomes were analyzed: EQ VAS scores and PROMIS Global Health raw sum scores. The EQ VAS was considered the primary outcome, reflecting respondents’ overall self-rated health, while PROMIS Global Health was included as a secondary outcome to provide a complementary multi-item measure of global health constructs. While recognizing that some bolt-ons may not correspond directly to the dimensions covered by PROMIS Global Health, the inclusion of both outcomes enabled an assessment of the incremental contribution of bolt-ons to overall HRQoL. Initially, univariable models were used to examine the association between each core dimension and bolt-on with the outcomes. Subsequently, multivariable models were built, beginning with the core dimensions and sequentially adding bolt-ons. Bolt-ons were added sequentially using a forward selection approach: at each step, the bolt-on that produced the greatest improvement in the model’s adjusted R^2^ was retained, and the process continued iteratively until the addition of further bolt-ons no longer improved model fit. The selection process was conducted separately for each country, allowing the sequence of retained bolt-ons to vary according to country-specific patterns of explanatory contribution. Both unadjusted and adjusted regression models, controlling for age and sex, were performed. All regression analyses were conducted at the item level to assess incremental explanatory contributions, independent of the latent structure identified in the PCA.

Bolt-ons were added sequentially in exploratory analyses to assess their incremental psychometric contribution; this approach was intended to evaluate cumulative effects rather than to suggest that multiple bolt-ons should be used simultaneously in practice. The RStudio with R version 4.5.0 (The R Foundation for Statistical Computing, Vienna, Austria) was used for all statistical analyses. PCA was conducted using the *psych* package [43].

## Results

### Characteristics of the study population

A total of 9025 participants were included in the final analysis, with 4,519 participants from China and 4506 participants from the Netherlands (Table 1). The Chinese sample had a mean age of 47 years, with 20% aged 65 or older, 47% identifying as female, and 66% residing in (sub)urban areas. In contrast, the Dutch sample had a mean age of 49 years, with 26% aged 65 or older, 55% identifying as female, and 90% residing in (sub)urban regions. While mean ages were similar between the two samples, the age distribution differed significantly, with a higher proportion of older respondents in the Dutch sample. In terms of health status, 56% of respondents in the Chinese sample and 69% in the Dutch sample reported having at least one chronic condition. The mean EQ VAS score was 80.2 (SD 16.7) in the Chinese sample and 73.6 (SD 19.4) in the Dutch sample.

**Table 1:** Demographic information of the study participants

Variables	CN	NL	*P*	Variables	CN	NL	*P*
Total (n (%))^*^	4519 (100)	4506 (100)					
Age (years)			< 0.001	Employment status			< 0.001
18–24	350 (7.7)	388 (8.6)		Employed	3062 (67.8)	2510 (55.7)	
25–34	763 (16.9)	749 (16.6)		Retired	1007 (22.3)	979 (21.7)	
35–44	1060 (23.5)	806 (17.9)		Unable to work due to disability or illness	20 (0.4)	372 (8.3)	
45–54	685 (15.2)	670 (14.9)		Student	144 (3.2)	187 (4.2)	
55–64	761 (16.8)	707 (15.7)		Unemployed	82 (1.8)	217 (4.8)	
65–74	884 (19.6)	843 (18.7)		Homemaker/housewife	146 (3.2)	147 (3.3)	
75–84	14 (0.3)	285 (6.3)		On maternity/paternity leave	2 (0)	8 (0.2)	
85 +	2 (0.04)	14 (0.3)		Other	0 (0)	72 (1.6)	
Missing	0 (0)	44 (1)		Missing	56 (1.2)	14 (0.3)	
Sex			< 0.001	Any chronic conditions	4411 (97.6)	4473 (99.3)	0.511^†^
Female	2142 (47.4)	2487 (55.2)		Hypertension	1035 (22.9)	914 (20.3)	
Male	2346 (51.9)	1977 (43.9)		Heart disease	166 (3.7)	378 (8.4)	
Missing	31 (0.7)	42 (0.9)		Stroke	70 (1.5)	109 (2.4)	
Highest level of education			< 0.001	Diabetes	330 (7.3)	439 (9.7)	
Primary school or less	639 (14.1)	306 (6.8)		Obesity	165 (3.7)	364 (8.1)	
Secondary school	934 (20.7)	952 (21.1)		Respiratory disease	263 (5.8)	514 (11.4)	
College/university degree or higher	2926 (64.7)	2573 (57.1)		Stomach ulcer	230 (5.1)	101 (2.2)	
Missing or decline to answer	20 (0.4)	675 (15)		Liver disease	119 (2.6)	52 (1.2)	
Marital status			< 0.001	Kidney disease	93 (2.1)	107 (2.4)	
Married	3714 (82.2)	1799 (39.9)		Gastrointestinal disease (e.g. IBD)	59 (1.3)	124 (2.8)	
Domestic partnership	72 (1.6)	677 (15)		IBS	59 (1.3)	311 (6.9)	
Single	557 (12.3)	1322 (29.3)		Rheumatoid arthritis	297 (6.6)	248 (5.5)	
Widowed	92 (2)	184 (4.1)		Osteoporosis	279 (6.2)	131 (2.9)	
Divorced/Legally separated	56 (1.2)	398 (8.8)		Other musculoskeletal disorders (muscle, tendon, nerve, or joint disorders)	62 (1.4)	423 (9.4)	
Other	11 (0.2)	116 (2.6)		Thyroid disease	102 (2.3)	194 (4.3)	
Missing	17 (0.4)	10 (0.2)		Skin diseases	342 (7.6)	355 (7.9)	
Place of residence			< 0.001	Cancer	16 (0.4)	241 (5.3)	
Urban (e.g. a large or small city)	2247 (49.7)	2544 (56.5)		Leukemia or lymphoma	23 (0.5)	26 (0.6)	
Suburban (e.g. in a suburb or on the edge of town)	736 (16.3)	1464 (32.5)		Anxiety	391 (8.7)	435 (9.7)	
Rural (e.g. in a village, on a farm or in a house in the countryside)	1513 (33.5)	438 (9.7)		Depression	177 (3.9)	674 (15)	
Missing	23 (0.5)	60 (1.3)		Eating disorders	80 (1.8)	110 (2.4)	
BMI			< 0.001	Other mental disorders	36 (0.8)	308 (6.8)	
Underweight	400 (8.9)	199 (4.4)		Sleep disorder	669 (14.8)	407 (9)	
Normal weight	3132 (69.3)	1893 (42)		Migraine	507 (11.2)	390 (8.7)	
Overweight	605 (13.4)	1469 (32.6)		Epilepsy	14 (0.3)	67 (1.5)	
Obese	277 (6.1)	904 (20.1)		Alzheimer's disease or dementia	0 (0)	12 (0.3)	
Missing	105 (2.3)	41 (0.9)		Other	25 (0.6)	531 (11.8)	
				None of the above	1858 (41.1)	1368 (30.4)	
				Missing	108 (2.4)	33 (0.7)	

^*^Percentages may not total 100 due to rounding

^†^*P*-value refers to the comparison between respondents with any chronic conditions

### Distributional characteristics

Ceiling, defined as reporting “no problems” on all core dimensions, was more pronounced in China than in the Netherlands (Table 2). In the Chinese dataset, 45% reported being in the best possible health state across all EQ-5D-5L dimensions, compared to 32% in the Dutch dataset. Across all bolt-ons, ceilings varied from 43% (vision) to 84% (breathing) in China, and from 37% (tiredness) to 79% (breathing) in the Netherlands. Among Chinese respondents, vision, tiredness, sleep, and cognition/memory, had lower ceilings than any of the five core dimensions. Among the Dutch respondents, only tiredness showed a lower ceiling compared to the core dimensions. The addition of bolt-ons to the EQ-5D-5L profile reduced the proportion of respondents reporting full health in both samples, with vision, tiredness, and sleep being the most effective. In China, ceilings decreased to 30% with vision, and to 35% with either tiredness or sleep. In the Netherlands, the corresponding figures were 22% (vision), 23% (tiredness), and 26% (sleep). In contrast, breathing had the least impact on reducing ceilings, with 43% in China and 30% in the Netherlands still reporting full health. This was followed by hearing in China (42%) and social relationships in the Netherlands (29%). The reduction in full-health ceiling became larger with the addition of more bolt-on items; however, the effect plateaued after the addition of the fourth bolt-on. Results from the propensity score matched sample showed similar distributions and ceilings for the bolt-ons across countries, consistent with the findings from the full sample (Appendices 4 and 5).

**Table 2:** Ceiling on EQ-5D-5L and bolt-ons

	Ceiling				Ceiling [EQ-5D-5L + bolt-on(s)]				Absolute ceiling reduction (%)		Relative ceiling reduction (%)	
	CN		NL		CN		NL					
	n	%	n	%	n	%	n	%	CN	NL	CN	NL
*EQ-5D-5L*												
Mobility	3785	83.8	3156	70.0	2022	44.7	1448	32.1	–	–	–	–
Self-care	4035	89.3	4037	89.6					–	–	–	–
Usual activities	3814	84.4	3064	68.0					–	–	–	–
Pain/discomfort	2747	60.8	2111	46.8					–	–	–	–
Anxiety/depression	2755	61.0	2831	62.8					–	–	–	–
*(EQ-5D-5L* +*) Bolt-on*												
Vision	1963	43.4	2252	50.0	1372	30.4	981	21.8	14.4	10.4	32.1	32.3
Tiredness	2096	46.4	1680	37.3	1579	34.9	1014	22.5	9.8	9.6	21.9	30.0
Sleep	2377	52.6	2353	52.2	1598	35.4	1148	25.5	9.4	6.7	21.0	20.7
Self-confidence	2947	65.2	2519	55.9	1765	39.1	1162	25.8	5.7	6.3	12.7	19.8
Hearing	3526	78.0	3407	75.6	1903	42.1	1262	28.0	2.6	4.1	5.9	12.8
Cognition/Memory	2647	58.6	2948	65.4	1692	37.4	1272	28.2	7.3	3.9	16.3	12.2
Social relationships	3164	70.0	3161	70.2	1789	39.6	1327	29.4	5.2	2.7	11.5	8.4
Breathing	3814	84.4	3538	78.5	1952	43.2	1364	30.3	1.5	1.9	3.5	5.8
*(EQ-5D-5L* +*) combinations of bolt-ons*												
TI + VI	1408	31.2	1061	23.5	1198	26.5	725	16.1	18.2	16.0	40.8	49.9
TI + VI + SL	1232	27.3	906	20.1	1097	24.3	660	14.6	20.5	17.5	45.7	54.4
TI + VI + SL + CO	1170	25.9	783	17.4	1050	23.2	590	13.1	21.5	19.0	48.1	59.3
TI + VI + SL + CG	1144	25.3	831	18.4	1036	22.9	622	13.8	21.8	18.3	48.8	57.0
TI + VI + SL + CO + HE	1147	25.4	707	15.7	1041	23.0	549	12.2	21.7	20.0	48.5	62.1
TI + VI + SL + CG + CO	1112	24.6	739	16.4	1010	22.4	564	12.5	22.4	19.6	50.0	61.0
All 8 bolt-ons	1058	23.4	633	14.0	974	21.6	509	11.3	23.2	20.8	51.8	64.8

CG, Cognition/memory; CN, China; CO, Self-confidence; HE, Hearing; NL, Netherlands; SL, Sleep; TI, Tiredness; VI, Vision

### Convergent and divergent validity between EQ-5D-5L and bolt-ons

Bolt-ons exhibited varying degrees of correlation with the core dimensions across both countries (Table 3). Overall, approximately 84% of the predefined hypotheses regarding convergent and divergent validity were confirmed across the two countries. Some exceptions to our hypotheses were observed. In China, vision and hearing, though at the lower bound of the moderate range, exhibited stronger correlations with some core dimensions than anticipated. Additionally, tiredness and sleep were only weakly correlated with usual activities, which was lower than expected. In the Netherlands, sleep and breathing showed weaker correlations with some core dimensions than anticipated.

**Table 3:** Convergent and divergent validity between EQ-5D-5L, bolt-ons, and EQ VAS

Variable^†*^		EQ-5D-5L					Bolt-ons							
		MO	SC	UA	PD	AD	VI	TI	SL	CO	HE	SR	BR	CG
*MO*	CN	–												
	NL	–												
*SC*	CN	0.63^***^	–											
	NL	0.39^*^	–											
*UA*	CN	0.66^***^	0.63^***^	–										
	NL	0.54^**^	0.44^**^	–										
*PD*	CN	0.47^**^	0.39^*^	0.44^**^	–									
	NL	0.55^**^	0.33^*^	0.57^**^	–									
*AD*	CN	0.31^*^	0.28^*^	0.33^*^	0.45^**^	–								
	NL	0.14	0.27^*^	0.35^*^	0.32^*^	–								
*VI*	CN	0.28^*^	0.23^*^	0.29^*^	0.41^**^	0.38^*^	–							
	NL	0.21^*^	0.17	0.24^*^	0.28^*^	0.19	–							
*TI*	CN	0.32^*^	0.27^*^	0.33^*^	0.51^**^	0.56^**^	0.47^**^	–						
	NL	0.31^*^	0.26^*^	0.50^**^	0.49^**^	0.52^**^	0.25^*^	–						
*SL*	CN	0.33^*^	0.27^*^	0.34^*^	0.44^**^	0.48^**^	0.43^**^	0.52^**^	–					
	NL	0.22^*^	0.23^*^	0.38^*^	0.38^*^	0.46^**^	0.24^*^	0.53^**^	–					
*CO*	CN	0.26^*^	0.26^*^	0.30^*^	0.34^*^	0.50^**^	0.39^*^	0.49^**^	0.44^**^	–				
	NL	0.13	0.20^*^	0.32^*^	0.27^*^	0.57^**^	0.18	0.46^**^	0.41^**^	–				
*HE*	CN	0.48^**^	0.47^**^	0.46^**^	0.44^**^	0.30^*^	0.36^*^	0.33^*^	0.34^*^	0.30^*^	–			
	NL	0.18	0.20^*^	0.19	0.22^*^	0.13	0.21^*^	0.16	0.16	0.13	–			
*SR*	CN	0.24^*^	0.25^*^	0.27^*^	0.28^*^	0.45^**^	0.35^*^	0.41^**^	0.38^*^	0.59^**^	0.27^*^	–		
	NL	0.15	0.25^*^	0.33^*^	0.24^*^	0.55^**^	0.19	0.40^**^	0.38^*^	0.53^**^	0.17	–		
*BR*	CN	0.40^**^	0.40^**^	0.41^**^	0.38^*^	0.36^*^	0.32^*^	0.33^*^	0.36^*^	0.35^*^	0.44^**^	0.33^*^	–	
	NL	0.25^*^	0.31^*^	0.35^*^	0.30^*^	0.27^*^	0.21^*^	0.34^*^	0.29^*^	0.24^*^	0.23^*^	0.27^*^	–	
*CG*	CN	0.37^*^	0.34^*^	0.38^*^	0.46^**^	0.42^**^	0.44^**^	0.47^**^	0.48^**^	0.46^**^	0.44^**^	0.41^**^	0.38^*^	–
	NL	0.23^*^	0.27^*^	0.39^*^	0.33^*^	0.41^**^	0.22^*^	0.46^**^	0.37^*^	0.43^**^	0.21^*^	0.40^**^	0.29^*^	–
*EQ VAS*	CN	−0.36^*^	−0.30^*^	−0.36^*^	−0.43^**^	−0.45^**^	−0.30^*^	−0.43^**^	−0.37^*^	−0.36^*^	−0.31^*^	−0.30^*^	−0.28^*^	−0.35^*^
	NL	−0.38^*^	−0.29^*^	−0.51^**^	−0.47^**^	−0.41^**^	−0.20^*^	−0.50^**^	−0.37^*^	−0.35^*^	−0.12	−0.32^*^	−0.28^*^	−0.33^*^

^*^ Indicates weak correlation strength (0.20–0.39)

^**^ Indicates moderate correlation strength (0.40–0.59)

^***^ Indicates strong correlation strength (≥ 0.60)

^**†**^ All correlation coefficients are statistically significant (*p* < 0.05)

AD, Anxiety/depression; BR, Breathing; CG, Cognition/memory; CN, China; CO, Self-confidence; HE, Hearing; MO, Mobility; NL, Netherlands; PD, Pain/discomfort; SC, Self-care; SLk, Sleep; SR, Social relationships; TI, Tiredness; UA, Usual activities; VI, Vision

### Convergent validity with external instruments

Convergent validity analyses largely supported the hypothesized relationships (Table 4). In most cases, correlations were stronger in the Dutch compared to Chinese samples. Tiredness demonstrated moderate-to-strong correlations with related items in both the WHO-5 and PROMIS instruments in the Netherlands (r = 0.55–0.69), while it showed weaker associations with both instruments in China (r = 0.38–0.54). Sleep showed moderate-to-strong correlations with PROMIS sleep disturbance items in both countries (CN: 0.42–0.58, NL: 0.46–0.67).

**Table 4:** Convergent validity of bolt-ons

HRQoL dimension^†^	Spearman’s correlation with EQ-5D-5L bolt-on^‡^	
	CN	NL
*Tiredness*		
EQ-5D-5L Tiredness bolt-on	–	–
WHO-5-feeling refreshed	0.38^*^	0.55^**^
PROMIS Global-Fatigue on average	0.54^**^	0.69^***^
*Sleep*		
EQ-5D-5L Sleep bolt-on	–	–
PROMIS Sleep quality-Sleep quality	0.58^**^	0.64^***^
PROMIS Sleep quality-Refreshing sleep	0.42^**^	0.46^**^
PROMIS Sleep quality-Problem with sleep	0.54^**^	0.67^***^
PROMIS Sleep quality-Difficulty falling asleep	0.54^**^	0.56^**^
*Social relationships*		
EQ-5D-5L Social relationships bolt-on	–	–
PROMIS Global-Satisfaction with social activities and relationships	0.43^**^	0.46^**^
PROMIS Global-Usual social activities and roles	0.35^*^	0.36^*^
ICECAP-A-Love, friendship, support	0.34^*^	0.43^**^
OPQOL^§^-Family, friends, neighbors	0.23^*^	0.25^*^
OPQOL^§^-Social, leisure activities	0.30^*^	0.29^*^
Social-Social safety net	0.27^*^	0.38^*^
*Cognition/memory*		
EQ-5D-5L Cognition/Memory bolt-on^||^	–	–
PROMIS Global-Mental health (incl. mood and ability to think)	0.40^**^	0.36^*^

^*^ Indicates weak correlation strength (0.20–0.39)

^**^ Indicates moderate correlation strength (0.40–0.59)

^***^ Indicates strong correlation strength (≥ 0.60)

^**†**^ All correlation coefficients are statistically significant (p < 0.05)

^‡^ Absolute values are reported

^§^ Completed only by respondents aged 65 + years

^||^ Worded as memory in China and cognition in Dutch; for wording refer to Appendix 1

CN, China; NL, Netherlands

Social relationships showed weak-to-moderate correlations with conceptually related items from external instruments, ranging from 0.23 to 0.43 in China and 0.25–0.46 in the Netherlands. Cognition/memory demonstrated a moderate correlation with its PROMIS counterpart in China (r = 0.40), but only a weak correlation in the Netherlands (r = 0.36). Overall, the pattern of correlations aligned with expectations in most cases, particularly for tiredness and sleep, supporting the convergent validity of these bolt-ons.

### Structural validity

PCA of the pooled dataset combining China and the Netherlands identified a seven-factor structure based on Kaiser’s criterion, accounting for 59% of the total variance (Table 5). The factors are presented in descending order of explained variance and represented the following latent constructs: general health, psychological functioning, positive wellbeing, physical functioning and pain, physical symptoms, sleep, and capability wellbeing. All eight bolt-ons loaded onto distinct factors separate from the core dimensions, suggesting that they capture aspects of HRQoL that may not be fully represented by the core dimensions. Vision, hearing, cognition/memory, and sleep demonstrated particularly strong loadings (standardized factor loadings ≥ 0.55), indicating a good fit with their respective latent constructs. Although social relationships and self-confidence are conceptually distinct from physical symptoms, they both loaded onto the physical symptoms factor.

**Table 5:** Pattern matrix of the PCA of CN and NL combined (promax rotation)

	Factors^*^						
	General health	Psychological functioning	Positive wellbeing	Physical functioning and pain	Physical symptoms	Sleep	Capability wellbeing
PROMIS Global-Health	0.901						
PROMIS Global-Physical health	0.886						
PROMIS Global-Quality of life	0.850						
PROMIS Global-Social health	0.849						
PROMIS Global-Discretionary social activities	0.821						
Social-Social safety net	0.766						
PROMIS Global-Mental health, incl. mood and ability to think	0.714						
PHQ-Feeling down, depressed, or hopeless		0.869					
GAD-Feeling nervous, anxious, or tense		0.859					
GAD-Cannot stop or control worries		0.811					
PHQ-Lack of interest or pleasure in activities		0.766					
PROMIS Global-Emotional problems, such as anxious, depressed, irritable		0.660					
EQ-5D-5L Anxiety/depression		0.481			0.448		
PROMIS Global-Fatigue on average		0.400					
WHO-5-Cheerful and in a good mood			0.904				
WHO-5-Calm and relaxed			0.898				
WHO-5-Active and purposeful			0.878				
WHO-5-Daily life is filled with things that interest oneself			0.866				
WHO-5-Refreshed and rested			0.806				
EQ-5D-5L Mobility (walking)				0.814			
PROMIS Global-Physical function				0.734			
EQ-5D-5L Usual activities (work, study, housework, family or leisure activities)				0.701			
EQ-5D-5L Self-care (washing or dressing)				0.679			
EQ-5D-5L Pain/discomfort				0.546			
PROMIS Global-Pain intensity numeric rating scale		0.343		0.526			
EQ-5D-5L Vision bolt-on					0.682		
EQ-5D-5L Hearing bolt-on					0.631		
EQ-5D-5L Social relationships bolt-on					0.613		
EQ-5D-5L Cognition/Memory bolt-on					0.586		
EQ-5D-5L Self confidence bolt-on					0.567		
EQ-5D-5L Breathing bolt-on (shortness of breath, wheezing, coughing, sputum)				0.304	0.528		
EQ-5D-5L Tiredness bolt-on					0.378		
PROMIS Sleep quality–Problem with sleep						0.819	
PROMIS Sleep quality–Difficulty falling asleep						0.763	
EQ-5D-5L Sleep bolt-on					0.424	0.706	
PROMIS Sleep quality–Refreshing sleep	0.337		0.306			0.409	
PROMIS Sleep quality–Sleep quality	0.465					0.395	0.705
ICECAP-A-Achievement and progress	0.434						0.508
ICECAP-A-Love, friendship, and support	0.355						0.478
ICECAP-A-Feeling at home and safe							0.467
ICECAP-A-Independence				0.332			0.453
ICECAP-A-Having fun and enjoying	0.376						0.383

^*^ Factor loadings >|0.3| are shown; absolute values are reported

Sensitivity analyses by country revealed generally consistent factor structures, with some notable differences (Appendices 6 and 7). Both country-specific analyses still supported a seven-factor structure, with slightly higher cumulative variance explained compared to the pooled dataset (CN: 60%, NL: 61%). In the Chinese dataset, hearing loaded onto the same factor as EQ-5D-5L dimensions. Conversely, anxiety/depression loaded onto the physical symptoms factor, aligning with most bolt-ons. In contrast, the Dutch dataset closely mirrored the factor structure observed in the pooled dataset. Hearing, vision, and sleep demonstrated particularly strong loadings in the Dutch dataset.

### Known-groups validity

Several bolt-ons increased the ability of EQ-5D-5L instrument to distinguish between subgroups of respondents (Table 6). In country-specific analyses, distinct patterns emerged. In China, hearing was especially effective in detecting age-related HRQoL decline, whereas in the Netherlands, self-confidence showed the largest gain in discrimination of HRQoL across age groups. Although all bolt-ons were initially considered for these analyses, the final models presented reflect country-specific statistical retention criteria. RE estimates are comparison-specific and should not be interpreted as absolute indicators of superiority across bolt-ons or health conditions. Similarly, cumulative gains observed when multiple bolt-ons were added are presented for exploratory purposes and do not imply recommendations regarding the routine inclusion of multiple bolt-ons.

**Table 6:** Known-groups validity of EQ-5D-5L and bolt-ons across age groups

Age group (years)	Mean (SD) EQ-5D-5L (+ bolt-on(s)) LSS (0–100)								RE (95%CI), ref: previous row	RE (95%CI), ref: no bolt-ons
	18–24	25–34	35–44	45–54	55–64	65–74	75–84	85 +		
CN (n (%))	350 (7.7)	763 (16.9)	1060 (23.5)	685 (15.2)	761 (16.8)	884 (19.6)	14 (0.3)	2 (0.04)		
EQ-5D-5L	4.3 (6.1)	4.8 (7.6)	4.8 (8.2)	5 (8.2)	8 (11.1)	13.2 (12.7)	20.7 (15.5)	37.5 (17.7)	–	–
EQ-5D-5L + HE	4 (5.7)	4.5 (7.3)	4.4 (7.8)	5 (8.1)	8.3 (11)	13.6 (12.7)	19.9 (14.7)	25.0 (-)	1.18 (1.1–1.4)	1.18 (1.1–1.4)
NL (n (%))	388 (8.6)	749 (16.6)	806 (17.9)	670 (14.9)	707 (15.7)	843 (18.7)	285 (6.3)	14 (0.3)		
EQ-5D-5L	12.4 (15.3)	13.1 (15.5)	13 (16.1)	12.3 (14.7)	14.4 (16.1)	12.4 (14.7)	12.9 (14.8)	19.2 (19.2)	–	–
EQ-5D-5L + CO	14.9 (15.3)	14.8 (15.8)	14.3 (16)	13.1 (14.6)	14.6 (15.7)	11.7 (13.4)	12.1 (13.6)	16.8 (16.9)	2.66 (1.03–5.17)	2.66 (1.03–5.17)
EQ-5D-5L + CO + SR	15.4 (15.4)	15.1 (15.7)	14.5 (16.3)	12.8 (14.2)	14 (15.3)	10.7 (12.5)	11 (12.3)	15.1 (14.6)	1.92 (1.44–2.28)	5.1 (1.72–10.21)
EQ-5D-5L + CO + SR + TI	17.9 (16.2)	17.6 (16.7)	16.9 (17.6)	15.3 (15.4)	16.4 (16.6)	12.1 (13.3)	12.4 (13.3)	16.7 (15.6)	1.38 (1.21–1.53)	7.03 (2.33–13.63)
EQ-5D-5L + CO + SR + TI + SL	19.2 (16.9)	18.9 (17.5)	17.7 (18.3)	16.3 (15.9)	17.3 (16.8)	12.6 (13.6)	12.9 (13.4)	16.3 (14.8)	1.15 (1.06–1.23)	8.08 (2.58–15.72)
EQ-5D-5L + CO + SR + TI + SL + CG	20 (17.8)	19.4 (18.1)	18.4 (19.3)	16.8 (16.3)	17.8 (17.3)	12.8 (13.6)	13 (13.5)	16.5 (14.8)	1.1 (1.04–1.16)	8.86 (2.81–17.58)

CG, Cognition/memory; CN, China; CO, Self-confidence; HE, Hearing; NL, Netherlands; SL, Sleep; SR, Social relationships; TI, Tiredness

In the Chinese participants, tiredness, self-confidence, sleep, cognition/memory, and vision improved the RE for assessing general health, quality of life, and physical health (Table 7). For mental health outcomes, tiredness, self-confidence, sleep, cognition/memory, and social relationships provided the largest gains in discriminative performance. In the Dutch participants, only tiredness improved the RE for assessing general and physical health. Meanwhile, self-confidence, tiredness, social relationships, and sleep enhanced the RE for quality of life and mental health.

**Table 7:** Known-groups validity of EQ-5D-5L and bolt-ons across health and quality of life groups

Health and quality of life	Mean (SD) EQ-5D-5L (+ bolt-on(s)) LSS (0–100)					RE (95%CI), ref: previous row	RE (95%CI), ref: no bolt-ons
Response levels	Poor	Fair	Good	Very good	Excellent		
*Health status (Global01)*							
CN (n (%))	58 (1.1)	592 (11.1)	1058 (19.8)	1701 (31.8)	1045 (36.3)	–	–
EQ-5D-5L	26.7 (18.72)	14.75 (11.29)	9.7 (10.81)	5.16 (8.4)	2.25 (5.37)	–	–
EQ-5D-5L + TI	29.02 (18.2)	17.42 (10.74)	11.72 (10.66)	6.55 (8.55)	2.99 (5.88)	1.3 (1.25–1.35)	1.3 (1.25–1.35)
EQ-5D-5L + TI + SL	30.42 (17.27)	18.85 (10.62)	12.98 (10.79)	7.41 (8.68)	3.33 (6.06)	1.13 (1.09–1.17)	1.47 (1.39–1.56)
EQ-5D-5L + TI + SL + CO	30.97 (16.32)	19.51 (10.47)	13.12 (10.64)	7.48 (8.66)	3.34 (5.99)	1.08 (1.05–1.11)	1.59 (1.49–1.7)
EQ-5D-5L + TI + SL + CO + CG	31.65 (16.45)	20.17 (10.46)	13.53 (10.69)	7.77 (8.68)	3.47 (6.05)	1.05 (1.03–1.07)	1.67 (1.55–1.8)
EQ-5D-5L + TI + SL + CO + CG + VI	32.1 (16.07)	21.04 (10.27)	14.62 (10.52)	8.68 (8.84)	4.05 (6.33)	1.03 (1.01–1.05)	1.72 (1.6–1.86)
NL (n (%))	205 (4.6)	1050 (23.3)	1660 (36.9)	1171 (26.0)	418 (9.3)	–	–
EQ-5D-5L	39.72 (18.91)	23.57 (15.69)	9.95 (11.06)	6.09 (9.94)	5.05 (11.98)	–	–
EQ-5D-5L + TI	43.98 (18.71)	26.92 (15.43)	12.2 (11.36)	7.74 (10.43)	6.09 (13.26)	1.16 (1.13–1.2)	1.16 (1.13–1.2)
*Quality of life (Global02)*							
CN (n (%))	79 (1.8)	734 (16.6)	1166 (26.3)	1535 (34.6)	918 (20.7)	–	–
EQ-5D-5L	17.17 (14.73)	13.15 (12.03)	9.05 (10.56)	4.81 (8.33)	2.89 (6.38)	–	–
EQ-5D-5L + TI	21 (14.63)	15.54 (11.64)	10.93 (10.48)	6.09 (8.55)	3.73 (6.85)	1.33 (1.27–1.4)	1.33 (1.27–1.4)
EQ-5D-5L + TI + CO	22.57 (14.28)	16.33 (11.39)	11.33 (10.42)	6.18 (8.35)	3.85 (6.99)	1.15 (1.11–1.2)	1.53 (1.43–1.65)
EQ-5D-5L + TI + CO + SL	24.25 (14.67)	17.41 (11.3)	12.26 (10.49)	6.81 (8.6)	4.27 (7.21)	1.08 (1.05–1.11)	1.65 (1.53–1.8)
EQ-5D-5L + TI + CO + SL + VI	24.41 (14.34)	18.53 (11.09)	13.53 (10.47)	7.78 (8.78)	4.97 (7.38)	1.04 (1.01–1.08)	1.73 (1.59–1.89)
EQ-5D-5L + TI + CO + SL + VI + CG	24.67 (14.35)	18.93 (11.21)	13.75 (10.51)	7.94 (8.85)	5.03 (7.4)	1.02 (1–1.04)	1.77 (1.62–1.95)
NL (n (%))	170 (3.8)	803 (17.9)	1642 (36.5)	1367 (30.4)	514 (11.4)	–	–
EQ-5D-5L	37.47 (20.52)	24.22 (17.35)	11.7 (12.23)	7.68 (11.41)	5.88 (10.88)	–	–
EQ-5D-5L + CO	39.75 (19.84)	25.5 (16.2)	12.34 (11.51)	8.16 (11.13)	5.97 (10.35)	1.23 (1.18–1.28)	1.23 (1.18–1.28)
EQ-5D-5L + CO + TI	44.87 (20.62)	29.53 (16.89)	14.78 (12.4)	9.95 (12.11)	7.07 (11.52)	1.12 (1.09–1.15)	1.38 (1.3–1.45)
EQ-5D-5L + CO + TI + SR	44.8 (20.38)	28.65 (16.12)	14.09 (11.84)	9.47 (11.93)	6.71 (10.99)	1.04 (1.01–1.06)	1.43 (1.34–1.53)
EQ-5D-5L + CO + TI + SR + SL	46.55 (20.41)	30.16 (16.5)	14.93 (12.13)	10.12 (12.46)	7.15 (11.37)	1.03 (1–1.05)	1.47 (1.37–1.58)
*Physical health (Global03)*							
CN (n (%))	92 (2.1)	694 (15.7)	1206 (27.2)	1549 (35.0)	887 (20.0)	–	–
EQ-5D-5L	22.56 (15.35)	14.61 (12.36)	8.92 (10.14)	4.32 (7.4)	2.11 (5.33)	–	–
EQ-5D-5L + TI	25.09 (14.63)	17.01 (11.91)	10.92 (9.98)	5.63 (7.74)	2.84 (5.89)	1.26 (1.22–1.32)	1.26 (1.22–1.32)
EQ-5D-5L + TI + SL	26.5 (13.71)	18.36 (11.79)	12.12 (10.15)	6.4 (7.94)	3.23 (6.14)	1.11 (1.07–1.15)	1.4 (1.33–1.48)
EQ-5D-5L + TI + SL + VI	27.2 (13.42)	19.57 (11.36)	13.65 (10.14)	7.51 (8.16)	3.93 (6.42)	1.06 (1.03–1.1)	1.49 (1.4–1.59)
EQ-5D-5L + TI + SL + VI + CO	27.4 (13.18)	19.74 (11.23)	13.62 (10)	7.5 (8.25)	3.89 (6.42)	1.02 (1–1.05)	1.53 (1.42–1.65)
NL (n (%))	254 (5.7)	1089 (24.2)	1649 (36.7)	1096 (24.4)	406 (9.0)	–	–
EQ-5D-5L	39.65 (18.07)	21.69 (15.24)	9.23 (10.75)	6.56 (10.69)	5.86 (12.48)	–	–
EQ-5D-5L + TI	43.92 (17.56)	24.71 (15.12)	11.53 (11.26)	8.2 (11.18)	7.15 (13.77)	1.13 (1.09–1.17)	1.13 (1.09–1.17)
*Mental health (Global04)*							
CN (n (%))	87 (2.0)	650 (14.6)	1112 (25.0)	1558 (35.1)	1034 (23.3)	–	–
EQ-5D-5L	16.88 (12.32)	14.37 (12.49)	9.18 (10.64)	5.05 (8.32)	2.65 (6.14)	–	–
EQ-5D-5L + CO	19.9 (11.76)	15.78 (11.76)	10 (10.49)	5.42 (8.07)	2.83 (6.24)	1.32 (1.26–1.39)	1.32 (1.26–1.39)
EQ-5D-5L + CO + TI	22.52 (11.77)	17.58 (11.57)	11.54 (10.55)	6.57 (8.33)	3.49 (6.63)	1.16 (1.13–1.2)	1.54 (1.44–1.64)
EQ-5D-5L + CO + TI + SL	24.41 (11.74)	18.72 (11.56)	12.49 (10.63)	7.18 (8.47)	3.88 (6.79)	1.1 (1.06–1.14)	1.69 (1.57–1.82)
EQ-5D-5L + CO + TI + SL + CG	25.59 (11.93)	19.35 (11.59)	12.88 (10.62)	7.44 (8.53)	4.03 (6.8)	1.06 (1.04–1.09)	1.8 (1.66–1.95)
EQ-5D-5L + CO + TI + SL + CG + SR	25.91 (12.09)	19.37 (11.41)	12.85 (10.63)	7.35 (8.48)	3.98 (6.71)	1.02 (1–1.05)	1.84 (1.69–2.01)
NL (n (%))	221 (4.9)	771 (17.1)	1512 (33.6)	1308 (29.1)	686 (15.3)	–	–
EQ-5D-5L	33.5 (17.88)	22.06 (17.19)	12.18 (13.5)	8.05 (11.98)	7.61 (11.99)	–	–
EQ-5D-5L + CO	37.82 (17.02)	23.75 (15.93)	12.74 (12.59)	8.22 (11.14)	7.39 (11.34)	1.47 (1.4–1.55)	1.47 (1.4–1.55)
EQ-5D-5L + CO + TI	42.82 (17.83)	27.59 (16.49)	15.3 (13.48)	9.97 (12.21)	8.7 (12.64)	1.13 (1.1–1.17)	1.66 (1.56–1.77)
EQ-5D-5L + CO + TI + SR	42.95 (17.52)	26.83 (15.85)	14.63 (12.85)	9.38 (11.76)	8.23 (12.2)	1.07 (1.04–1.1)	1.78 (1.65–1.92)
EQ-5D-5L + CO + TI + SR + SL	44.63 (17.79)	28.29 (16.14)	15.6 (13.28)	10.01 (12.18)	8.58 (12.57)	1.04 (1.02–1.07)	1.85 (1.7–2.01)

CG, Cognition/memory; CN, China; CO, self-confidence; NL, Netherlands; SL, Sleep; SR, Social relationships; TI, Tiredness; VI, Vision

The inclusion of specific bolt-ons increased discrimination between participants with and without chronic conditions (Table 8). The RE of the bolt-on-enhanced EQ-5D-5L versions ranged from 1.06 (95% CI: 1.00–1.12) to 2.27 (95% CI: 1.63–3.93) in China, and 1.12 (95% CI: 1.04–1.23) to 1.87 (95% CI: 1.53–2.38) in the Netherlands. Several bolt-ons consistently demonstrated strong performance across both countries for specific conditions. Breathing improved discrimination in respiratory and cardiovascular disease, while tiredness was particularly informative in IBS. Sleep added value in distinguishing individuals with headache/migraine and sleep disorders, and self-confidence showed strong performance in anxiety, depression, and other mental health disorders. Differences in the bolt-on combinations retained across countries and conditions reflect variations in statistically significant incremental discrimination among the candidate bolt-ons that were specified *a priori* based on predefined hypotheses.

**Table 8:** Known-groups validity of EQ-5D-5L and bolt-ons across health condition groups

EQ-5D-5L + selected bolt-on(s)	EQ-5D-5L (+ bolt-on(s)) ceiling (%)		EQ-5D-5L (+ bolt-on(s)) mean (SD) LSS		RE (95%CI), ref: previous row	RE (95%CI), ref: no bolt-ons
	Healthy (CN: n = 1858; NL: n = 1368)	Chronic condition	Healthy (CN: n = 1858; NL: n = 1368)	Chronic condition		
*Respiratory disease (e.g. asthma, COPD)*						
CN (n = 263)						
EQ-5D-5L	68.8	17.9	2.7 (5.5)	14.4 (15.3)	–	–
EQ-5D-5L + BR	67.5	15.6	2.4 (5.1)	14.9 (15.2)	1.38 (1.24–1.57)	1.38 (1.24–1.57)
EQ-5D-5L + BR + TI	56.6	9.1	3.4 (5.7)	16.8 (14.7)	1.08 (1.01–1.19)	1.50 (1.30–1.77)
NL (n = 514)						
EQ-5D-5L	59.5	13.2	4.7 (8.4)	21 (18.4)	–	–
EQ-5D-5L + BR	57.8	8.2	4.3 (7.7)	21.8 (17.2)	1.75 (1.57–1.99)	1.75 (1.57–1.99)
*Hypertension*						
CN (n = 1035)						
EQ-5D-5L	68.8	26.4	2.7 (5.5)	11.5 (12.2)	–	–
EQ-5D-5L + BR	67.5	24.3	2.4 (5.1)	11 (11.7)	1.06 (1–1.12)	1.06 (1–1.12)
NL (n = 914)						
EQ-5D-5L	59.5	23.9	4.7 (8.4)	16.7 (17.2)	–	–
*Cardiovascular diseases*						
CN (n = 166)						
EQ-5D-5L	68.8	26.5	2.7 (5.5)	14 (14.1)	–	–
EQ-5D-5L + BR	67.5	25.3	2.4 (5.1)	13.7 (13.9)	1.12 (1.01–1.26)	1.12 (1.01–1.26)
NL (n = 378)						
EQ-5D-5L	59.5	17.7	4.7 (8.4)	19.3 (18.1)	–	–
EQ-5D-5L + BR	57.8	16.9	4.3 (7.7)	18.7 (17.7)	1.29 (1.15–1.48)	1.29 (1.15–1.48)
*Osteoporosis*						
CN (n = 279)						
EQ-5D-5L	68.8	22.9	2.7 (5.5)	13.1 (13.6)	–	–
EQ-5D-5L + TI	57.3	13.6	3.8 (6.2)	15.1 (13.7)	1.11 (1–1.26)	1.11 (1–1.26)
NL (n = 131)						
EQ-5D-5L	59.5	10.7	4.7 (8.4)	27.5 (21)	–	–
*Diabetes*						
CN (n = 330)						
EQ-5D-5L	68.8	25.8	2.7 (5.5)	12 (12.1)	–	–
EQ-5D-5L + SL	58.7	16.7	3.4 (5.8)	13.9 (12.2)	1.23 (1.08–1.43)	1.23 (1.08–1.43)
NL (n = 439)						
EQ-5D-5L	59.5	21.0	4.7 (8.4)	18.5 (17.6)	–	–
*Stomach ulcer*						
CN (n = 230)						
EQ-5D-5L	68.8	19.1	2.7 (5.5)	10.8 (10.1)	–	–
EQ-5D-5L + CO	61.9	15.2	3.3 (6)	12.3 (10.6)	1.47 (1.21–1.91)	1.47 (1.21–1.91)
EQ-5D-5L + CO + TI	53.8	10.9	4.2 (6.6)	14.1 (10.7)	1.20 (1.07–1.40)	1.77 (1.40–2.51)
NL (n = 101)						
EQ-5D-5L	59.5	13.9	4.7 (8.4)	25 (20.8)	–	–
EQ-5D-5L + SR	55.3	11.9	4.9 (8.2)	25.4 (20.8)	1.2 (1.03–1.42)	1.2 (1.03–1.42)
*GI disease*						
CN (n = 59)						
EQ-5D-5L	68.8	25.4	2.7 (5.5)	17.1 (19.8)	–	–
NL (n = 124)						
EQ-5D-5L	59.5	17.7	4.7 (8.4)	23.7 (22.1)	–	–
EQ-5D-5L + BR	57.8	15.3	4.3 (7.7)	22.7 (20.9)	1.16 (1–1.38)	1.16 (1–1.38)
*IBS*						
CN (n = 59)						
EQ-5D-5L	68.8	28.8	2.7 (5.5)	10.4 (12.9)	–	–
EQ-5D-5L + TI	57.3	16.9	3.8 (6.2)	13 (13.5)	1.6 (1.11–7.87)	1.6 (1.11–7.87)
NL (n = 311)						
EQ-5D-5L	59.5	9.3	4.7 (8.4)	23.2 (18.1)	–	–
EQ-5D-5L + TI	42.6	5.5	6.6 (9.3)	26.6 (18.5)	1.12 (1.04–1.23)	1.12 (1.04–1.23)
*Headache, migraine*						
CN (n = 507)						
EQ-5D-5L	68.8	21.1	2.7 (5.5)	11.8 (11.6)	–	–
EQ-5D-5L + SL	58.7	13.8	3.4 (5.8)	13.8 (11.4)	1.22 (1.1–1.38)	1.22 (1.1–1.38)
NL (n = 390)						
EQ-5D-5L	59.5	16.2	4.7 (8.4)		–	–
EQ-5D-5L + SL	49.9	9.2	5.7 (8.7)	20.7 (16.8)	1.35 (1.17–1.61)	1.35 (1.17–1.61)
EQ-5D-5L + SL + TI	39.2	6.4	7.1 (9.5)	23.3 (17.6)	1.14 (1.05–1.27)	1.54 (1.29–1.94)
*Stroke*						
CN (n = 70)						
EQ-5D-5L	68.8	17.1	2.7 (5.5)	21.1 (19.8)	–	–
NL (n = 109)						
EQ-5D-5L	59.5	15.6	4.7 (8.4)	24.8 (20.9)	–	–
EQ-5D-5L + BR	57.8	11.0	4.3 (7.7)	24.4 (20.1)	1.26 (1.09–1.52)	1.26 (1.09–1.52)
EQ-5D-5L + BR + CG	52.9	8.3	4.5 (7.6)	25.1 (19.5)	1.17 (1.02–1.39)	1.47 (1.18–1.96)
*Anxiety*						
CN (n = 391)						
EQ-5D-5L	68.8	13.8	2.7 (5.5)	13.6 (11.6)	–	–
EQ-5D-5L + CO	61.9	9.5	3.3 (6)	15.1 (11.5)	1.31 (1.18–1.46)	1.31 (1.18–1.46)
EQ-5D-5L + CO + TI	53.8	5.6	4.2 (6.6)	17.1 (11.2)	1.17 (1.09–1.26)	1.53 (1.33–1.77)
EQ-5D-5L + CO + TI + SL	49.2	4.9	4.5 (6.7)	18.5 (11.4)	1.1 (1.04–1.16)	1.68 (1.43–2)
NL (n = 435)						
EQ-5D-5L	59.5	8.3	4.7 (8.4)	24.9 (17.8)	–	–
EQ-5D-5L + CO	49.4	3.4	5.6 (8.5)	27.8 (17.8)	1.51 (1.4–1.65)	1.51 (1.4–1.65)
EQ-5D-5L + CO + SR	47.0	3.0	5.7 (8.4)	28.3 (17.9)	1.16 (1.1–1.22)	1.75 (1.57–1.97)
EQ-5D-5L + CO + SR + TI	36.6	2.3	7.2 (9.4)	31.9 (18.7)	1.05 (1.01–1.09)	1.83 (1.62–2.09)
*Depression*						
CN (n = 177)						
EQ-5D-5L	68.8	15.8	2.7 (5.5)	13.8 (12.8)	–	–
EQ-5D-5L + CO	61.9	9.6	3.3 (6)	15.8 (12.9)	1.45 (1.24–1.75)	1.45 (1.24–1.75)
EQ-5D-5L + CO + SR	59.4	7.3	3.6 (6.4)	16.8 (12.7)	1.17 (1.05–1.34)	1.69 (1.36–2.26)
EQ-5D-5L + CO + SR + SL	52.6	5.1	4 (6.4)	18.3 (13)	1.11 (1.03–1.21)	1.88 (1.48–2.56)
NL (n = 674)						
EQ-5D-5L	59.5	7.9	4.7 (8.4)	23.5 (17.6)	–	–
EQ-5D-5L + CO	49.4	4.5	5.6 (8.5)	26 (17.4)	1.49 (1.39–1.61)	1.49 (1.39–1.61)
EQ-5D-5L + CO + SR	47.0	4.2	5.7 (8.4)	26.4 (17.5)	1.17 (1.12–1.22)	1.74 (1.58–1.94)
EQ-5D-5L + CO + SR + TI	36.6	2.4	7.2 (9.4)	29.9 (18.3)	1.06 (1.03–1.1)	1.86 (1.67–2.07)
*Skin disease*						
CN (n = 342)						
EQ-5D-5L	68.8	25.4	2.7 (5.5)	9.7 (11)	–	–
EQ-5D-5L + TI	57.3	16.1	3.8 (6.2)	12.1 (11.3)	1.64 (1.33–2.36)	1.64 (1.33–2.36)
EQ-5D-5L + TI + CO	53.8	12.6	4.2 (6.6)	12.8 (11.3)	1.23 (1.07–1.49)	2.02 (1.49–3.35)
EQ-5D-5L + TI + CO + SL	49.2	10.8	4.5 (6.7)	13.9 (11.6)	1.13 (1.01–1.28)	2.27 (1.63–3.93)
NL (n = 355)						
EQ-5D-5L	59.5	14.4	4.7 (8.4)	20 (17.9)	–	–
*Sleep disorder*						
CN (n = 669)						
EQ-5D-5L	68.8	19.7	2.7 (5.5)	12.3 (12.1)	–	–
EQ-5D-5L + SL	58.7	9.1	3.4 (5.8)	15.5 (11.8)	1.79 (1.63–2.03)	1.79 (1.63–2.03)
EQ-5D-5L + SL + TI	51.9	6.9	4.2 (6.4)	17.1 (11.7)	1.07 (1.02–1.13)	1.92 (1.71–2.22)
NL (n = 407)						
EQ-5D-5L	59.5	8.8	4.7 (8.4)	25.7 (18.3)	–	–
EQ-5D-5L + SL	49.9	2.5	5.7 (8.7)	30.3 (17.9)	1.73 (1.58–1.91)	1.73 (1.58–1.91)
*Thyroid disease*						
CN (n = 102)						
EQ-5D-5L	68.8	31.4	2.7 (5.5)	7.8 (9.9)	–	–
NL (n = 194)						
EQ-5D-5L	59.5	16.0	4.7 (8.4)	18.8 (17.2)	–	–
EQ-5D-5L + CG	54.2	11.9	4.9 (8.1)	19.3 (16.9)	1.28 (1.07–1.63)	1.28 (1.07–1.63)
*Eating disorders*						
CN (n = 80)						
EQ-5D-5L	68.8	26.3	2.7 (5.5)	15.3 (17.3)	–	–
NL (n = 110)						
EQ-5D-5L	59.5	8.2	4.7 (8.4)	26.7 (19.2)	–	–
EQ-5D-5L + CO	49.4	5.5	5.6 (8.5)	30.4 (19.3)	1.59 (1.37–1.92)	1.59 (1.37–1.92)
EQ-5D-5L + CO + SR	47.0	4.5	5.7 (8.4)	31.2 (20)	1.18 (1.08–1.28)	1.87 (1.53–2.38)
*Other mental disorders*						
CN (n = 36)						
EQ-5D-5L	68.8	30.6	2.7 (5.5)	13.7 (14.7)	–	–
EQ-5D-5L + CO	61.9	25.0	3.3 (6)	15.9 (16.1)	1.50 (1.10–2.74)	1.50 (1.10–2.74)
NL (n = 308)						
EQ-5D-5L	59.5	7.8	4.7 (8.4)	25.8 (18.3)	–	–
EQ-5D-5L + CO	49.4	3.9	5.6 (8.5)	28.4 (18.1)	1.40 (1.29–1.54)	1.40 (1.29–1.54)
EQ-5D-5L + CO + SR	47.0	3.6	5.7 (8.4)	29.2 (18.1)	1.20 (1.13–1.28)	1.68 (1.49–1.92)
*Obesity*						
CN (n = 165)						
EQ-5D-5L	68.8	35.2	2.7 (5.5)	9.4 (12.4)	–	–
EQ-5D-5L + SR	62.7	27.9	3.1 (5.8)	10.4 (12.4)	1.68 (1.21–5.43)	1.68 (1.21–5.43)
NL (n = 364)						
EQ-5D-5L	59.5	12.4	4.7 (8.4)	24.1 (19.6)	–	–

BR, Breathing; CG, Cognition/memory; CO, Self-confidence; SL, Sleep; SR, Social relationships; TI, Tiredness

Sensitivity analysis adjusting for age and sex produced results that were broadly consistent with the unadjusted known-groups validity findings (Appendix 8). In most cases, improvements in discrimination between chronic condition groups plateaued after the inclusion of a maximum of two bolt-ons. After adjustment, most chronic conditions continued to show significant differences across both countries. However, hypertension was no longer significant in either China or the Netherlands. Across conditions, breathing and sleep consistently demonstrated relatively strong discriminatory performance in both countries. Overall, these findings suggest that the known-groups validity results are robust to adjustment for age and sex.

### Explanatory power

Univariable linear regression analyses showed that the bolt-ons varied considerably in their explanatory strength (Table 9). In China, tiredness yielded the highest adjusted R^2^ for EQ VAS (0.14), with vision contributing the least (0.07). In the Netherlands, tiredness again showed the highest explanatory power (0.24), while hearing performed the weakest (0.02). A similar pattern emerged for PROMIS Global Health, where tiredness had the strongest individual explanatory value in both countries (CN 0.22, NL: 0.26). In contrast, the weakest associations differed by country, with breathing showing the lowest explanatory power in China (0.05) and hearing in the Netherlands (0.01). Multivariable linear regression analyses adjusted for age and sex showed that EQ-5D-5L explained a smaller proportion of variance in PROMIS Global Health and EQ VAS scores in China (0.23–0.25), compared to the Netherlands (0.29–0.37). The addition of bolt-ons in the adjusted multivariable models incrementally increased the proportion of explained variance, with tiredness and self-confidence providing the largest contributions. These adjusted R^2^ gains should be interpreted comparatively within this study context rather than as absolute measures of predictive power. Likewise, cumulative improvements observed with the addition of multiple bolt-ons are exploratory and should not be interpreted as recommendations for routine use of multiple bolt-ons.

**Table 9:** Uni- and multivariable regressions on EQ VAS and PROMIS Global Health total score

Selection of dimensions	CN				NL			
	EQ VAS		PROMIS Global Health total score (raw)		EQ VAS		PROMIS Global Health total score (raw)	
	Adjusted R^2^	ΣΔ adjusted R^2^	Adjusted R^2^	ΣΔ adjusted R^2^	Adjusted R^2^	ΣΔ adjusted R^2^	Adjusted R^2^	ΣΔ adjusted R^2^
*EQ-5D-5L dimensions*								
Mobility (MO)	0.1268	–	0.0637	–	0.1498	–	0.0712	–
Self–care (SC)	0.0995	–	0.0458	–	0.0944	–	0.0332	–
Usual activities (UA)	0.1206	–	0.0645	–	0.2852	–	0.1773	–
Pain/discomfort (PD)	0.1476	–	0.1362	–	0.2301	–	0.0846	–
Anxiety/depression (AD)	0.1715	–	0.1966	–	0.1727	–	0.2014	–
*Bolt-on dimensions*								
Vision (VI)	0.0660	–	0.1360	–	0.0371	–	0.0314	–
Tiredness (TI)	0.1420	–	0.2153	–	0.2402	–	0.2635	–
Sleep (SL)	0.1206	–	0.1780	–	0.1516	–	0.1474	–
Self-confidence (CO)	0.1080	–	0.1974	–	0.1321	–	0.2129	–
Hearing (HE)	0.0942	–	0.0611	–	0.0158	–	0.0058	–
Social relationships (SR)	0.0732	–	0.1532	–	0.1180	–	0.1696	–
Breathing (BR)	0.0722	–	0.0534	–	0.0806	–	0.0369	–
Cognition/Memory (CG)	0.1158	–	0.1779	–	0.1256	–	0.1227	–
*EQ-5D-5L (*+ bolt-on(s)) dimensions^*^								
EQ-5D-5L	0.2468	–	0.2314	–	0.3728	–	0.2942	–
EQ-5D-5L + TI	–	–	–	–	0.3872	0.0144	0.3487	0.0545
EQ-5D-5L + CO	0.2574	0.0106	0.2868	0.0554	–	–	–	–
EQ-5D-5L + TI + CO	0.2644	0.0176	0.3098	0.0784	0.3909	0.0181	0.3813	0.0871
EQ-5D-5L + TI + CO + SL	0.2672	0.0204	–	–	0.3931	0.0202	–	–
EQ-5D-5L + TI + CO + SR	–	–	–	–	–	–	0.3889	0.0947
EQ-5D-5L + TI + CO + CG	–	–	0.3238	0.0924	–	–	–	–
EQ-5D-5L + TI + CO + SL + VI	0.2690	0.0223	–	–	0.3951	0.0222	–	–
EQ-5D-5L + TI + CO + SR + HE	–	–	–	–	–	–	0.3942	0.1000
EQ-5D-5L + TI + CO + CG + VI	–	–	0.3342	0.1028	–	–	–	–
EQ-5D-5L + TI + CO + SL + VI + BR	0.2696	0.0228	–	–	0.3960	0.0231	–	–
EQ-5D-5L + TI + CO + SR + HE + SL	–	–	–	–	–	–	0.3970	0.1028
EQ-5D-5L + TI + CO + CG + VI + SL			0.3401	0.1087	–	–	–	–
EQ-5D-5L + TI + CO + SL + VI + CG + SR	–	–	0.3444	0.1130	0.3966	0.0238	–	–
EQ-5D-5L + TI + CO + SR + HE + SL + BR	–	–	–	–	–	–	0.3988	0.1046
EQ-5D-5L + TI + CO + SL + VI + BR + CG	0.2697	0.0229	–	–	–	–	–	–
EQ-5D-5L + TI + CO + SL + VI + CG + SR + BR	–	–	0.3492	0.1177	–	–	–	–
EQ-5D-5L + TI + CO + SL + VI + CG + SR + BR + HE	–	–	0.3511	0.1196	–	–	–	–

^*^Adjusted for age and sex

CN, China; NL, Netherlands

## Discussion

This study presented a comprehensive psychometric evaluation of eight EQ-5D-5L bolt-ons using large quota-based samples approximating the adult population of China and the Netherlands. Leveraging robust data from the EQ-DAPHNIE project [26, 27], we applied a wide range of analytical techniques to assess different forms of validity of each bolt-on.

At the profile level, vision, tiredness, and sleep bolt-ons consistently reduced ceilings. In contrast, tiredness and self-confidence demonstrated stronger construct-specific discrimination in known-groups analyses across health status groups and contributed to the largest increases in explained variance in models predicting EQ VAS and PROMIS Global Health scores. Importantly, profile-level ceilings should be distinguished from dimension-level ceilings, which reflect the prevalence of “no problems” responses on individual dimensions. While reduced profile-level ceilings indicate greater informational spread in healthier respondents, they should not be interpreted as direct evidence of improved construct validity. For example, although breathing exhibited high dimension-level ceiling in the general population, it still showed strong known-groups discrimination among respondents with relevant chronic conditions. This illustrates that a high ceiling at the population level does not necessarily imply low discriminative value when the dimension is applied to clinically relevant subgroups.

Although this study examined the incremental effects of adding multiple bolt-ons, these analyses were conducted for exploratory and comparative purposes rather than to advocate the simultaneous use of numerous bolt-ons in applied settings. In practice, it has been suggested that the inclusion of one or two bolt-ons tailored to a specific patient population is most feasible and appropriate [20]. The observed cumulative improvements in ceiling reductions and explanatory power when additional bolt-ons were included are therefore expected and primarily serve to illustrate the extent to which different HRQoL dimensions contribute uniquely beyond the core EQ-5D-5L dimensions. Additionally, the explanatory power analyses should be interpreted considering the broad scope of PROMIS Global Health. While suitable as a global health measure, it may not fully align with all bolt-on dimensions, potentially limiting the explanatory contribution of certain dimensions.

Bolt-ons showed robust psychometric properties in both countries, although the observed patterns varied by population subgroup and country. Importantly, these differences should not be interpreted as direct comparisons of bolt-on performance between countries, as they may reflect variation in health distributions, reporting behavior, cultural factors, translation, and other contextual factors. While the overall prevalence of chronic conditions was similar, the distribution of specific conditions differed, with higher prevalence of mental health and musculoskeletal conditions in the Netherlands. Such differences in morbidity profiles may partly explain cross-country variations in the performance of bolt-ons related to psychosocial functioning, such as self-confidence and social relationships.

An additional consideration concerns the cognition/memory bolt-on. The Dutch survey assessed a broader cognition construct encompassing memory, comprehension, concentration, and thinking, whereas the Chinese survey assessed memory only. This difference represents a potential lack of conceptual equivalence and may therefore affect both construct validity and cross-country comparability. Consequently, observed differences involving this bolt-on should be interpreted cautiously, as they may reflect differences in the underlying construct being measured rather than true differences in psychometric properties.

It is important to note that the RE in known-groups analyses is contrast-dependent and should be interpreted within each predefined comparison rather than as a global measure across all bolt-ons. Large RE gains observed after sequentially adding multiple bolt-ons reflect exploratory cumulative explanatory improvements and do not imply a recommendation to include many bolt-ons simultaneously. Finally, although chronic condition subgroups with at least 30 participants were included in the known-groups analysis, some subgroups remained small, which may contribute to wide confidence intervals and should be considered when interpreting these findings.

While some bolt-ons, such as tiredness and sleep, performed consistently across countries, others showed context-specific variation. Notably, tiredness exhibited a higher ceiling in China than in the Netherlands, despite higher labor force participation in the Chinese sample and commonly perceived differences in work-life balance between the two countries. This finding may reflect cross-cultural differences in the perception, normalization, or reporting of tiredness, rather than differences in actual tiredness levels [44]. Previous research suggests that cultural context can influence how HRQoL is conceptualized and reported, with Western measures often emphasizing psychological and individual well-being, whereas studies conducted in East Asian contexts have highlighted additional culturally specific health dimensions and differences in emotional reporting and health perception [18, 45–47]. In some contexts, tiredness may be viewed as a normal part of daily life and therefore less likely to be reported as a health problem, whereas in other settings, it may be more readily recognized and reported as an impairment. Interestingly, vision showed lower ceiling in China, which may reflect a higher prevalence of visual impairments in China [48]. Similarly, hearing provided stronger discrimination across age groups in China, while self-confidence and social relationships were more informative in the Netherlands. These patterns may align with previously described cross-cultural differences in HRQoL perception, where functional and sensory health dimensions may be more salient in some East Asian contexts [18]. The stronger age-group discrimination observed for the hearing bolt-on in China may partly reflect population-specific factors, such as differences in hearing impairment prevalence associated with age, place of residence, access to hearing aids, or occupational exposure, which cannot be fully disentangled from cultural or reporting effects in the present study. Importantly, the present study was not designed to disentangle cultural, linguistic, methodological, and epidemiological influences on the observed differences. Consequently, these findings highlight the importance of considering local health perceptions, reporting behavior, and population characteristics when testing and interpreting bolt-on performance across countries.

These cross-country differences should also be interpreted in the broader context of how bolt-ons are typically used. While the present study was conducted in general population samples from China and the Netherlands, bolt-ons are primarily intended for use in specific patient populations. In addition, bolt-ons are often applied in multi-country clinical studies where the use of different bolt-ons across countries would generally be avoided to preserve comparability of results. Therefore, these findings provide insights into how additional health dimensions may vary in their measurement properties across populations and may help inform the selection and evaluation of bolt-ons in future research and population-based studies.

Although bolt-ons such as vision, tiredness, and sleep showed consistently strong psychometric properties, others demonstrated more modest psychometric properties. For example, the social relationships bolt-on generally showed weak correlations with external instruments, likely reflecting the broader and context-dependent nature of this construct. Similarly, bolt-ons such as breathing and cognition/memory exhibited high ceilings in general population samples, limiting their incremental contribution outside specific subgroups. These findings emphasize the importance of selecting bolt-ons that are relevant to study population and research objectives.

PCA suggested that the bolt-ons did not load on the same factors as the core dimensions, indicating that they capture additional aspects of health not fully represented by the core dimensions. Some latent factors, such as “physical symptoms”, included both physical and psychosocial bolt-ons (self-confidence, social relationships); these labels should be interpreted cautiously and not viewed as definitive conceptual constructs. The results also suggested a potential methods effect, as bolt-ons tended to cluster according to their originating instruments rather than strictly by conceptual dimensions [49]. This pattern may reflect shared item characteristics, such as wording, response scales, recall periods, or placement within the survey, rather than substantive differences in underlying constructs [50]. Importantly, the PCA was conducted primarily to demonstrate the separability of bolt-ons from core dimensions, rather than to define latent constructs. While this limits the extent to which individual factors can be interpreted purely as conceptual constructs, the analysis confirms that bolt-ons provide distinct measurement information beyond the core EQ-5D-5L dimensions. Future studies could adopt multitrait-multimethod frameworks to explicitly model both trait and method effects, examining relationships at the scale level [51].

There are several limitations of this study. First, the use of online panel-based, non-probability sampling may have introduced selection bias. Although quota sampling was applied to approximate population distributions, participation was limited to individuals who are digitally literate, have access to the internet, and may be more inclined to engage with health-related surveys. Second, respondents with the most severe levels of health impairment were underrepresented, potentially limiting the evaluation of bolt-ons across the full spectrum of health severity. Third, the EQ-5D-5L and bolt-ons were administered in a fixed order, which may have introduced context or ordering effects. However, the order of the external measures was randomized to mitigate potential ordering bias. Fourth, the translation of bolt-on items involved forward translation from English into Dutch and Simplified Chinese without cognitive debriefing [26], except for the Dutch self-confidence item, which was available in a forward–backward translated and cognitively debriefed version. Fifth, the wording of the cognition/memory bolt-on differed across the two countries, potentially contributing to the observed differences in psychometric properties. Lastly, the cross-sectional design precluded assessment of test–retest reliability and responsiveness.

## Conclusions

This study provides evidence that several EQ-5D-5L bolt-ons demonstrate robust psychometric properties in general population samples from China and the Netherlands. While bolt-ons such as tiredness, sleep, vision and self-confidence enhanced the discriminatory power and explanatory capacity of the EQ-5D-5L, the observed patterns varied across countries and population subgroups. These findings should therefore be interpreted within their specific cultural, linguistic, and methodological contexts. Rather than identifying universally preferred bolt-ons, the results contribute to the ongoing development of the EQ-5D Bolt-on Toolbox by providing evidence on the validity and properties of individual bolt-ons across different settings. The findings highlight the importance of context-specific validation and may help inform bolt-on selection for future population health surveys and clinical studies.

## Supplementary Information

Below is the link to the electronic supplementary material.

Supplementary Material 1

## Data Availability

Data can be obtained from the EuroQol Research Foundation’s Storage and Access for Valuable EuroQol Data (SAVED) archive.
