# Supplementary material for: Comparative performance of EQ-5D-5L bolt-ons in China and the Netherlands: results from the EQ-DAPHNIE project
Source: Qual Life Res. 2026 Jun 28;35(8):222. doi: 10.1007/s11136-026-04328-3 (PMC13310819; doi:10.1007/s11136-026-04328-3)
Supplement: Supplementary file 1 — Supplementary Material 1 [file 11136_2026_4328_MOESM1_ESM.docx]

**Comparative Performance of EQ-5D-5L Bolt-ons in China and the Netherlands: Results from the EQ-DAPHNIE project**

Authors:

Seo-Ho Cho^1,2,3,4^, Mathieu F. Janssen^5^, Fatima Al Sayah^6^, Zhuxin Mao^7^, You-Shan Feng^8^, Lajos V. Kemény^2,3,4,9,10^, Fanni Rencz^2,11,12^ *on behalf of the EQ-DAPHNIE project team*

**Appendices**

**Appendix 1 Wording of the EQ-5D-5L bolt-ons used in EQ-DAPHNIE in China and in the Netherlands**

**Appendix 2 Additional outcome measures**

**Appendix 3 Chronic conditions and corresponding prespecified EQ-5D-5L bolt-ons**

**Appendix 4 Bolt-on response distribution of all respondents and propensity score matched samples**

**Appendix 5 Ceiling on EQ-5D-5L and bolt-ons of propensity score matched samples**

**Appendix 6 Pattern matrix of the PCA of CN (promax rotation)**

**Appendix 7 Pattern matrix of the PCA of NL (promax rotation)**

**Appendix 8 Adjusted known-group validity of EQ-5D-5L and bolt-ons across health condition groups**

**Appendix 1. Wording of the EQ-5D-5L bolt-ons used in EQ-DAPHNIE in China and in the Netherlands**

| **Dimension heading** | **Breathing problems** | **Cognition (memory, comprehension, concentration, thinking)** *(used in the NL)* | **Memory** *(used in China)* | **Vision (using glasses or contact lenses if needed)** | **Hearing (using equipment if needed, e.g. hearing aids)** |
| --- | --- | --- | --- | --- | --- |
| Level 1 | I have no breathing problems | I have no problems with cognition | I have no problems remembering | I have no problems seeing | I have no problems hearing |
| Level 2 | I have slight breathing problems | I have slight problems with cognition | I have slight problems remembering | I have slight problems seeing | I have slight problems hearing |
| Level 3 | I have moderate breathing problems | I have moderate problems with cognition | I have moderate problems remembering | I have moderate problems seeing | I have moderate problems hearing |
| Level 4 | I have severe breathing problems | I have severe problems with cognition | I have severe problems remembering | I have severe problems seeing | I have severe problems hearing |
| Level 5 | I have extreme breathing problems | I have extreme problems with cognition | I have extreme problems remembering | I have extreme problems seeing | I have extreme problems hearing |
| **Dimension heading** | **Sleep** | **Tiredness** | **Self-confidence** | **Social relationships** |  |
| Level 1 | I have no problems sleeping | I am not tired | I have no problems with self-confidence | I have no problems with social relationships |  |
| Level 2 | I have slight problems sleeping | I am slightly tired | I have slight problems with self-confidence | I have slight problems with social relationships |  |
| Level 3 | I have moderate problems sleeping | I am moderately tired | I have moderate problems with self-confidence  SAMPLE | I have moderate problems with social relationships |  |
| Level 4 | I have severe problems sleeping | I am severely tired | I have severe problems with self-confidence | I have severe problems with social relationships |  |
| Level 5 | I have extreme problems sleeping | I am extremely tired | I have extreme problems with self-confidence | I have extreme problems with social relationships |  |

***© EuroQol Research Foundation. EQ-5D^TM^ is a trade mark of the EuroQol Research Foundation. This is a modified EQ-5D. Reproduced by permission of EuroQol Research Foundation. Reproduction of this version is not allowed. For reproduction, use or modification of the EQ-5D (any version), please register your study by using the online EQ registration page:*** [***www.euroqol.org***](http://www.euroqol.org)

**Appendix 2. Additional outcome measures**

*PROMIS Global Health and PROMIS Sleep Disturbance Short Form*

PROMIS Global Health is a brief, standardized assessment of general health status [1]. It consists of 10 items drawn from the broader PROMIS (Patient-Reported Outcomes Measurement Information System) framework. Nine items are rated using a 5-point Likert scale, while one item, pain intensity, is rated on an 11-point numeric scale ranging from 0 (“no pain”) to 10 (“worst imaginable pain”). Three items use a recall period of “the past 7 days”, whereas the remaining items assess health status “in general”. The PROMIS captures multiple domains including physical, mental, and social health.

*PROMIS Sleep Disturbance Short Form* is designed to assess the quality of sleep and the presence of sleep-related problems [2]. It includes items that evaluate sleep-related issues such as sleep quality, sleep disturbances, and difficulty falling asleep over the past 7 days. The responses are measured on a 5-point Likert scale, with higher scores indicating greater sleep disturbance.

*ICEpop CAPability measure for Adults (ICECAP-A)*

ICECAP-A is a standardized assessment used to measure individuals’ capabilities to do and be things they value in life. It consists of five attributes: stability, attachment, autonomy, achievement, and enjoyment [3]. Each dimension is rated on a four-level scale, ranging from 1 (“not being able to experience a capability at all”) to 4 (“being able to fully experience a capability”). The recall period for all items is “at the moment”, reflecting on the individuals’ current state of capability.

*WHO-5 well-being index*

The WHO-5 well-being index is a standardized assessment of subjective mental well-being over the past two weeks [4]. The scale consists of five positively worded statements on a six-point Likert scale ranging from 0 (“at no time”) to 5 (“all of the time”).

*Older People’s Quality of Life questionnaire-brief (OPQOL-brief)*

The OPQOL-brief is a standardized assessment of quality of life among older adults. It consists of 14 items and can be grouped into two dimensions: ‘psychological well-being’ and ‘life restrictions and limitations’. Each item is rated on a 5-point Likert scale [5]. The questionnaire was administered using adaptive logic so that only participants aged 65 years and older were presented with the OPQOL-brief [6].

*2-item Patient Health Questionnaire (PHQ-2) and 2-item Generalized Anxiety Disorder questionnaire (GAD-2)*

The PHQ-2 and GAD-2 are standardized assessments for symptoms of depression and anxiety, respectively, over the last two weeks [7, 8]. The PHQ-2 includes two items assessing the presence and frequency of depressive symptoms, while the GAD-2 includes two items measuring the presence and frequency of anxiety symptoms. Each item in both instruments is rated on a 4-point scale ranging from 0 (“not at all”) to 3 (“nearly every day”).

*Social determinants of health*

Measures related to social determinants of health were also included, which were partly adapted from the European Social Survey Source Questionnaire Development [9]. These items captured aspects of social connection and perceived social safety net. One item asked “Which of the following best describes your safety net?”, with responses rated on a 5-point scale ranging from 1 (“excellent”) to 5 (“poor”).

**Appendix 3. Chronic conditions and corresponding prespecified EQ-5D-5L bolt-ons**

| **Disease** [10]**^*^** | **Vision** | **Hearing** | **Breathing** | **Sleep** | **Tiredness** | **Social relationships** | **Self-confidence** | **Cognition** |
| --- | --- | --- | --- | --- | --- | --- | --- | --- |
| Respiratory disease [11-14] |  |  | X | X | X | X | X |  |
| Hypertension [15-21] |  |  | X | X | X | X | X |  |
| Cardiovascular disease [15-17, 21-24] |  |  | X | X | X | X | X | X |
| Osteoporosis [25-28] |  |  |  | X | X | X | X |  |
| Diabetes [18, 29-36] | X |  | X | X | X | X | X |  |
| Stomach ulcer [37-42] |  |  | X | X | X | X | X |  |
| GI disease [37, 38, 42-45] |  |  | X | X | X | X | X |  |
| IBS [46-53] |  |  | X | X | X | X | X |  |
| Headache, migraine [54-60] |  |  |  | X | X | X | X | X |
| Stroke [61-69] | X |  | X | X | X | X | X | X |
| Anxiety [70, 71] |  |  |  | X | X | X | X | X |
| Depression [72-74] |  |  |  | X | X | X | X | X |
| Skin diseases [75-81] |  |  |  | X | X | X | X |  |
| Sleep disorder [82-86] |  |  |  | X | X | X | X | X |
| Thyroid disease [87-92] | X |  |  | X | X | X | X | X |
| Eating disorders [93-99] |  |  |  | X | X | X | X | X |
| Other mental disorders [100, 101] |  |  |  | X | X | X | X | X |
| Obesity [18, 102-105] |  |  | X | X | X | X | X |  |

**^*^** Based on the literature and the authors’ expert opinion

**Appendix 4 Bolt-on response distribution of all respondents and propensity score matched samples**

1. Bolt-on response distribution of all respondents (CN: n=4519; NL: n=4506)

1. Bolt-on response distribution of propensity score matched samples (CN: n=4425; NL: n=4425)

**Appendix 5 Ceiling on EQ-5D-5L and bolt-ons of propensity score matched samples**

|  | **Ceiling** | | | | **Ceiling [EQ-5D-5L + bolt-on(s)]** | | | | **Absolute ceiling reduction (%)** | | **Relative ceiling reduction (%)** | |
| --- | --- | --- | --- | --- | --- | --- | --- | --- | --- | --- | --- | --- |
|  | **CN** | | **NL** | | **CN** | | **NL** | |  |  |  |  |
|  | **n** | **%** | **n** | **%** | **n** | **%** | **n** | **%** | **CN** | **NL** | **CN** | **NL** |
| **EQ-5D-5L** |  |  |  |  |  |  |  |  |  |  |  |  |
| Mobility | 3703 | 83.7 | 3106 | 70.2 | 1983 | 44.8 | 1423 | 32.2 | - | - | - | - |
| Self-care | 3948 | 89.2 | 3968 | 89.7 |  |  |  |  | - | - | - | - |
| Usual activities | 3735 | 84.4 | 3013 | 68.1 |  |  |  |  | - | - | - | - |
| Pain/discomfort | 2683 | 60.6 | 2079 | 47.0 |  |  |  |  | - | - | - | - |
| Anxiety/depression | 2706 | 61.2 | 2771 | 62.6 |  |  |  |  | - | - | - | - |
| **(EQ-5D-5L +) Bolt-on** |  |  |  |  |  |  |  |  |  |  |  |  |
| Vision | 1921 | 43.4 | 2216 | 50.1 | 1347 | 30.4 | 965 | 21.8 | 14.4 | 10.4 | 32.1 | 32.2 |
| Tiredness | 2056 | 46.5 | 1650 | 37.3 | 1551 | 35.1 | 996 | 22.5 | 9.8 | 9.6 | 21.8 | 30.0 |
| Sleep | 2328 | 52.6 | 2305 | 52.1 | 1568 | 35.4 | 1130 | 25.5 | 9.4 | 6.6 | 20.9 | 20.6 |
| Self-confidence | 2888 | 65.3 | 2468 | 55.8 | 1729 | 39.1 | 1139 | 25.7 | 5.7 | 6.4 | 12.8 | 20.0 |
| Hearing | 3449 | 77.9 | 3351 | 75.7 | 1867 | 42.2 | 1242 | 28.1 | 2.6 | 4.1 | 5.8 | 12.7 |
| Cognition | 2595 | 58.6 | 2892 | 65.4 | 1656 | 37.4 | 1251 | 28.3 | 7.4 | 3.9 | 16.5 | 12.1 |
| Social relationships | 3098 | 70.0 | 3097 | 70.0 | 1751 | 39.6 | 1304 | 29.5 | 5.2 | 2.7 | 11.7 | 8.4 |
| Breathing problems | 3737 | 84.5 | 3479 | 78.6 | 1917 | 43.3 | 1342 | 30.3 | 1.5 | 1.8 | 3.3 | 5.7 |
| **(EQ-5D-5L +) combinations of bolt-ons** |  |  |  |  |  |  |  |  |  |  |  |  |
| TI+VI | 1382 | 31.2 | 1041 | 23.5 | 1177 | 26.6 | 711 | 16.1 | 18.2 | 16.1 | 40.6 | 50.0 |
| TI+VI+SL | 1212 | 27.4 | 889 | 20.1 | 1078 | 24.4 | 648 | 14.6 | 20.5 | 17.5 | 45.6 | 54.5 |
| TI+VI+SL+CO | 1151 | 26.0 | 767 | 17.3 | 1032 | 23.3 | 578 | 13.1 | 21.5 | 19.1 | 48.0 | 59.4 |
| TI+VI+SL+CG | 1124 | 25.4 | 816 | 18.4 | 1017 | 23.0 | 611 | 13.8 | 21.8 | 18.4 | 48.7 | 57.1 |
| TI+VI+SL+CO+HE | 1129 | 25.5 | 694 | 15.7 | 1024 | 23.1 | 539 | 12.2 | 21.7 | 20.0 | 48.4 | 62.1 |
| TI+VI+SL+CG+CO | 1093 | 24.7 | 724 | 16.4 | 992 | 22.4 | 553 | 12.5 | 22.4 | 19.7 | 50.0 | 61.1 |
| All 8 bolt-ons | 1041 | 23.5 | 623 | 14.1 | 958 | 21.6 | 501 | 11.3 | 23.2 | 20.8 | 51.7 | 64.8 |

CG: cognition; CN: China; CO: self-confidence; HE: hearing; NL: Netherlands; SL: sleep; TI: tiredness; VI: vision

**Appendix 6. Pattern matrix of the PCA of CN (promax rotation)**

|  | **Factors**^*^**^†^** | | | | | | |
| --- | --- | --- | --- | --- | --- | --- | --- |
|  | **General health** | **Physical functioning and pain** | **Positive wellbeing** | **Psychological functioning** | **Sleep** | **Capability wellbeing** | **Physical symptoms** |
| PROMIS Global - Social health | 0.891 |  |  |  |  |  |  |
| PROMIS Global - Physical health | 0.888 |  |  |  |  |  |  |
| PROMIS Global - Health | 0.865 |  |  |  |  |  |  |
| PROMIS Global - Quality of life | 0.855 |  |  |  |  |  |  |
| PROMIS Global - Mental health, incl. mood and ability to think | 0.825 |  |  |  |  |  |  |
| PROMIS Global - Discretionary social activities | 0.824 |  |  |  |  |  |  |
| Social - Social safety net | 0.635 |  |  |  |  |  |  |
| **EQ-5D-5L Mobility (walking)** |  | 0.895 |  |  |  |  |  |
| **EQ-5D-5L Self-care (washing or dressing)** |  | 0.890 |  |  |  |  |  |
| **EQ-5D-5L Usual activities (work, study, housework, family or leisure activities)** |  | 0.852 |  |  |  |  |  |
| **EQ-5D-5L Hearing bolt-on** |  | 0.655 |  |  |  |  |  |
| PROMIS Global - Physical function |  | 0.601 |  |  |  |  | 0.320 |
| **EQ-5D-5L Pain/discomfort** |  | 0.461 |  |  |  |  |  |
| WHO5-5 - Cheerful and in a good mood |  |  | 0.861 |  |  |  |  |
| WHO5-5 - Calm and relaxed |  |  | 0.876 |  |  |  |  |
| WHO5-5 - Active and purposeful |  |  | 0.837 |  |  |  |  |
| WHO5-5 - Daily life is filled with things that interest oneself |  |  | 0.828 |  |  |  |  |
| WHO-5 - Refreshed and rested |  |  | 0.803 |  |  |  |  |
| PHQ -Feeling down, depressed, or hopeless |  |  |  | 0.779 |  |  |  |
| GAD - Feeling nervous, anxious, or tense |  |  |  | 0.740 |  |  |  |
| PHQ - Lack of interest or pleasure in activities |  |  |  | 0.685 |  |  |  |
| GAD - Cannot stop or control worries |  |  |  | 0.681 |  |  |  |
| PROMIS Global - Emotional problems, such as anxious, depressed, irritable |  |  |  | 0.570 |  |  |  |
| PROMIS Global - Fatigue on average |  |  |  | 0.444 |  |  |  |
| PROMIS Global - Pain intensity 0-10 |  | 0.306 |  | 0.390 |  |  |  |
| PROMIS Sleep quality - Problem with sleep |  |  |  |  | 0.944 |  |  |
| PROMIS Sleep quality - Difficulty falling asleep |  |  |  |  | 0.940 |  |  |
| PROMIS Sleep quality - Sleep quality | 0.306 |  |  |  | 0.684 |  |  |
| **EQ-5D-5L Sleep bolt-on** |  |  |  |  | 0.678 |  | 0.407 |
| PROMIS Sleep quality - Refreshing sleep |  |  |  |  | 0.504 |  |  |
| ICECAP-A - Achievement and progress |  |  |  |  |  | 0.840 |  |
| ICECAP-A - Feeling at home and safe |  |  |  |  |  | 0.807 |  |
| ICECAP-A - Love, friendship, and support |  |  |  |  |  | 0.768 |  |
| ICECAP-A - Having fun and enjoying |  |  |  |  |  | 0.650 |  |
| ICECAP-A - Independence |  |  |  |  |  | 0.624 |  |
| **EQ-5D-5L Social relationships bolt-on** |  |  |  |  |  |  | 0.755 |
| **EQ-5D-5L Self confidence bolt-on** |  |  |  |  |  |  | 0.711 |
| **EQ-5D-5L Vision bolt-on** |  |  |  |  |  |  | 0.609 |
| **EQ-5D-5L Anxiety/depression** |  |  |  | 0.360 |  |  | 0.554 |
| **EQ-5D-5L Tiredness bolt-on** |  |  |  |  |  |  | 0.527 |
| **EQ-5D-5L Memory** |  |  |  |  |  |  | 0.477 |
| **EQ-5D-5L Breathing bolt-on (shortness of breath, wheezing, coughing, sputum)** |  | 0.437 |  |  |  |  | 0.455 |

^*^ Factor loadings >|0.3| are shown; absolute values are reported

**^†^** Factors are presented in descending order of explained variance.

**Appendix 7. Pattern matrix of the PCA of NL (promax rotation)**

|  | **Factors**^*^**^†^** | | | | | | |
| --- | --- | --- | --- | --- | --- | --- | --- |
|  | **General health** | **Physical functioning and pain** | **Positive wellbeing** | **Psychological functioning** | **Sleep** | **Capability wellbeing** | **Physical symptoms** |
| PROMIS Global - Quality of life | 0.802 |  |  |  |  |  |  |
| PROMIS Global - Physical health | 0.793 | 0.351 |  |  |  |  |  |
| PROMIS Global - Health | 0.781 | 0.311 |  |  |  |  |  |
| PROMIS Global - Discretionary social activities | 0.747 |  |  |  |  |  |  |
| PROMIS Global - Social health | 0.738 |  |  |  |  |  |  |
| PROMIS Global - Mental health, incl. mood and ability to think | 0.663 |  |  |  |  |  |  |
| Social - Social safety net | 0.527 |  |  |  |  | 0.410 |  |
| **EQ-5D-5L Mobility (walking)** |  | 0.759 |  |  |  |  |  |
| PROMIS Global - Physical function |  | 0.759 |  |  |  |  |  |
| PROMIS Global - Pain intensity 0-10 |  | 0.631 |  | 0.399 |  |  |  |
| **EQ-5D-5L Usual activities (work, study, housework, family or leisure activities)** |  | 0.626 |  |  |  |  |  |
| **EQ-5D-5L Pain/discomfort** |  | 0.594 |  |  |  |  |  |
| **EQ-5D-5L Self-care (washing or dressing)** |  | 0.532 |  |  |  |  |  |
| WHO5-5 - Cheerful and in a good mood |  |  | 0.905 |  |  |  |  |
| WHO5-5 - Active and purposeful |  |  | 0.900 |  |  |  |  |
| WHO5-5 - Calm and relaxed |  |  | 0.890 |  |  |  |  |
| WHO5-5 - Daily life is filled with things that interest oneself |  |  | 0.873 |  |  |  |  |
| WHO-5 - Refreshed and rested |  |  | 0.800 |  |  |  |  |
| PHQ -Feeling down, depressed, or hopeless |  |  |  | 0.895 |  |  |  |
| GAD - Feeling nervous, anxious, or tense |  |  |  | 0.897 |  |  |  |
| GAD - Cannot stop or control worries |  |  |  | 0.880 |  |  |  |
| PHQ - Lack of interest or pleasure in activities |  |  |  | 0.795 |  |  |  |
| PROMIS Global - Emotional problems, such as anxious, depressed, irritable |  |  |  | 0.744 |  |  |  |
| **EQ-5D-5L Anxiety/depression** |  |  |  | 0.527 |  |  | 0.324 |
| PROMIS Global - Fatigue on average |  |  |  | 0.454 |  |  |  |
| PROMIS Sleep quality - Problem with sleep |  |  |  |  | 0.904 |  |  |
| PROMIS Sleep quality - Sleep quality |  |  |  |  | 0.844 |  |  |
| PROMIS Sleep quality - Difficulty falling asleep |  |  |  |  | 0.818 |  |  |
| **EQ-5D-5L Sleep bolt-on** |  |  |  |  | 0.815 |  |  |
| PROMIS Sleep quality - Refreshing sleep |  |  | 0.337 |  | 0.579 |  |  |
| ICECAP-A - Love, friendship, and support |  |  |  |  |  | 0.799 |  |
| ICECAP-A - Achievement and progress |  |  |  |  |  | 0.726 |  |
| ICECAP-A - Independence |  | 0.375 |  |  |  | 0.701 |  |
| ICECAP-A - Feeling at home and safe |  |  |  |  |  | 0.640 |  |
| ICECAP-A - Having fun and enjoying |  |  |  |  |  | 0.611 |  |
| **EQ-5D-5L Hearing bolt-on** |  |  |  |  |  |  | 0.721 |
| **EQ-5D-5L Vision bolt-on** |  |  |  |  |  |  | 0.693 |
| **EQ-5D-5L Breathing bolt-on (shortness of breath, wheezing, coughing, sputum)** |  |  |  |  |  |  | 0.513 |
| **EQ-5D-5L Cognition bolt-on (memory, comprehension, concentration, thinking)** |  |  |  |  |  |  | 0.511 |
| **EQ-5D-5L Social relationships bolt-on** |  |  |  |  |  | 0.352 | 0.400 |
| **EQ-5D-5L Tiredness bolt-on** |  |  |  |  |  |  | 0.371 |
| **EQ-5D-5L Self confidence bolt-on** |  |  |  |  |  |  | 0.364 |

^*^ Factor loadings >|0.3| are shown; absolute values are reported

**^†^** Factors are presented in descending order of explained variance.

**Appendix 8 Adjusted known-group validity of EQ-5D-5L and bolt-ons across health condition groups**

| **EQ-5D-5L + selected bolt-on(s)** | **Mean (95% CI) EQ-5D-5L (+bolt-on(s)) LSS (0-100)** | | **p-value** | **Cohen's d** |
| --- | --- | --- | --- | --- |
|  | **Healthy (CN: n=1858; NL: n=1368)** | **Chronic condition** |  |  |
| **Respiratory disease (e.g. asthma, COPD)** |  |  |  |  |
| **CN (n=263)** |  |  |  |  |
| EQ-5D-5L | 2.73 (2.39-3.08) | 13.94 (13.01-14.86) | <0.001 | 1.51 (1.37-1.66) |
| EQ-5D-5L+BR | 2.46 (2.13-2.79) | 14.42 (13.53-15.32) | <0.001 | 1.69 (1.54-1.83) |
| EQ-5D-5L+BR+SL | 3.12 (2.79-3.46) | 16.04 (15.13-16.95) | <0.001 | 1.79 (1.65-1.94) |
| EQ-5D-5L+BR+SL+TI | 3.89 (3.54-4.24) | 17.70 (16.70-18.60) | <0.001 | 1.83 (1.69-1.98) |
| **NL (n=514)** |  |  |  |  |
| EQ-5D-5L | 4.67 (4.02-5.32) | 20.97 (19.88-22.06) | <0.001 | 1.36 (1.24-1.48) |
| EQ-5D-5L+BR | 4.24 (3.63-4.84) | 21.80 (20.78-22.82) | <0.001 | 1.58 (1.46-1.69) |
| EQ-5D-5L+BR+SL | 5.04 (4.42-5.66) | 23.10 (22.05-24.14) | <0.001 | 1.59 (1.47-1.71) |
| **Cardiovascular diseases** |  |  |  |  |
| **CN (n=166)** |  |  |  |  |
| EQ-5D-5L | 2.74 (2.43-3.05) | 13.19 (12.12-14.26) | <0.001 | 1.59 (1.41-1.76) |
| EQ-5D-5L+BR | 2.47 (2.18-2.76) | 12.89 (11.88-13.90) | <0.001 | 1.68 (1.50-1.85) |
| EQ-5D-5L+BR+SL | 3.13 (2.83-3.43) | 14.20 (13.16-15.24) | <0.001 | 1.74 (1.56-1.92) |
| EQ-5D-5L+BR+SL+CG | 3.56 (3.25-3.87) | 15.10 (14.00-16.20) | <0.001 | 1.75 (1.57-1.93) |
| **NL (n=378)** |  |  |  |  |
| EQ-5D-5L | 4.54 (3.92-5.15) | 19.94 (18.72-21.16) | <0.001 | 1.37 (1.24-1.50) |
| EQ-5D-5L+BR | 4.08 (3.50-4.67) | 19.42 (18.26-20.58) | <0.001 | 1.44 (1.30-1.57) |
| EQ-5D-5L+BR+CG | 4.22 (3.65-4.80) | 19.66 (18.53-20.80) | <0.001 | 1.48 (1.34-1.61) |
| **Osteoporosis** |  |  |  |  |
| **CN (n=279)** |  |  |  |  |
| EQ-5D-5L | 2.76 (2.43-3.09) | 12.49 (11.58-13.40) | <0.001 | 1.38 (1.23-1.52) |
| EQ-5D-5L+SL | 3.48 (3.14-3.81) | 14.14 (13.21-15.07) | <0.001 | 1.48 (1.33-1.63) |
| EQ-5D-5L+SL+TI | 4.29 (3.93-4.65) | 15.89 (14.90-16.89) | <0.001 | 1.51 (1.36-1.66) |
| **NL (n=131)** |  |  |  |  |
| EQ-5D-5L | 4.64 (4.09-5.19) | 27.58 (25.72-29.44) | <0.001 | 2.25 (2.04-2.46) |
| **Diabetes** |  |  |  |  |
| **CN (n=330)** |  |  |  |  |
| EQ-5D-5L | 2.78 (2.46-3.11) | 11.40 (10.60-12.20) | <0.001 | 1.25 (1.12-1.38) |
| EQ-5D-5L+SL | 3.51 (3.18-3.84) | 13.45 (12.63-14.28) | <0.001 | 1.40 (1.27-1.54) |
| EQ-5D-5L+SL+BR | 3.17 (2.85-3.49) | 12.76 (11.98-13.54) | <0.001 | 1.43 (1.29-1.56) |
| EQ-5D-5L+SL+BR+VI | 4.23 (3.90-4.56) | 14.40 (13.50-15.20) | <0.001 | 1.45 (1.32-1.59) |
| **NL (n=439)** |  |  |  |  |
| EQ-5D-5L | 4.49 (3.87-5.11) | 19.23 (18.10-20.37) | <0.001 | 1.30 (1.18-1.43) |
| EQ-5D-5L+BR | 4.02 (3.45-4.60) | 18.11 (17.06-19.17) | <0.001 | 1.34 (1.22-1.47) |
| **Stomach ulcer** |  |  |  |  |
| **CN (n=230)** |  |  |  |  |
| EQ-5D-5L | 2.70 (2.42-2.99) | 10.63 (9.81-11.46) | <0.001 | 1.29 (1.14-1.43) |
| EQ-5D-5L+SL | 3.43 (3.13-3.73) | 12.62 (11.76-13.47) | <0.001 | 1.44 (1.29-1.59) |
| EQ-5D-5L+SL+BR | 3.10 (2.82-3.37) | 12.00 (11.20-12.80) | <0.001 | 1.49 (1.34-1.64) |
| EQ-5D-5L+SL+BR+TI | 3.87 (3.57-4.17) | 13.70 (12.90-.60) | <0.001 | 1.52 (1.37-1.67) |
| **NL (n=101)** |  |  |  |  |
| EQ-5D-5L | 4.67 (4.14-5.19) | 25.84 (23.88-27.79) | <0.001 | 2.17 (1.95-2.4) |
| EQ-5D-5L+SR | 4.90 (4.38-5.41) | 26.30 (24.39-28.20) | <0.001 | 2.25 (2.03-2.47) |
| EQ-5D-5L+SR+BR | 4.52 (4.04-5.00) | 24.62 (22.85-26.39) | <0.001 | 2.28 (2.05-2.50) |
| **GI disease** |  |  |  |  |
| **CN (n=59)** |  |  |  |  |
| EQ-5D-5L | 2.68 (2.38-2.97) | 16.84 (15.15-18.53) | <0.001 | 2.22 (1.94-2.50) |
| EQ-5D-5L+BR | 2.41 (2.14-2.69) | 16.72 (15.14-18.29) | <0.001 | 2.41 (2.13-2.69) |
| EQ-5D-5L+BR+SL | 3.08 (2.79-3.36) | 18.38 (16.75-20.00) | <0.001 | 2.50 (2.22-2.78) |
| **NL (n=124)** |  |  |  |  |
| EQ-5D-5L | 4.67 (4.12-5.22) | 23.81 (21.96-25.66) | <0.001 | 1.86 (1.66-2.06) |
| EQ-5D-5L+BR | 4.26 (3.75-4.77) | 22.95 (21.24-24.67) | <0.001 | 1.96 (1.76-2.16) |
| EQ-5D-5L+BR+TI | 5.93 (5.37-6.48) | 26.09 (24.25-27.94) | <0.001 | 1.97 (1.76-2.17) |
| **IBS** |  |  |  |  |
| **CN (n=59)** |  |  |  |  |
| EQ-5D-5L | 2.67 (2.4-2.94) | 10.17 (8.62-11.72) | <0.001 | 1.28 (1.01-1.55) |
| EQ-5D-5L+TI | 3.78 (3.47-4.08) | 12.81 (11.08-14.54) | <0.001 | 1.38 (1.11-1.65) |
| EQ-5D-5L+TI+BR | 3.39 (3.11-3.68) | 12.21 (10.61-13.82) | <0.001 | 1.45 (1.18-1.72) |
| EQ-5D-5L+TI+BR+SL | 3.85 (3.56-4.14) | 13.00 (11.30-14.60) | <0.001 | 1.47 (1.20-1.74) |
| **NL (n=311)** |  |  |  |  |
| EQ-5D-5L | 4.68 (4.09-5.27) | 23.01 (21.73-24.29) | <0.001 | 1.68 (1.54-1.82) |
| EQ-5D-5L+SL | 5.60 (5.01-6.19) | 24.30 (23.03-25.58) | <0.001 | 1.72 (1.58-1.86) |
| EQ-5D-5L+SL+TI | 7.05 (6.43-7.67) | 26.99 (25.64-28.35) | <0.001 | 1.73 (1.59-1.87) |
| EQ-5D-5L+SL+TI+BR | 6.44 (5.86-7.01) | 24.90 (23.60-26.10) | <0.001 | 1.74 (1.59-1.88) |
| **Headache, migraine** |  |  |  |  |
| **CN (n=507)** |  |  |  |  |
| EQ-5D-5L | 2.79 (2.46-3.12) | 11.46 (10.82-12.10) | <0.001 | 1.21 (1.10-1.31) |
| EQ-5D-5L+SL | 3.51 (3.18-3.85) | 13.38 (12.73-14.03) | <0.001 | 1.35 (1.24-1.46) |
| EQ-5D-5L+SL+TI | 4.33 (3.98-4.69) | 14.92 (14.23-15.61) | <0.001 | 1.38 (1.27-1.49) |
| **NL (n=390)** |  |  |  |  |
| EQ-5D-5L | 4.67 (4.09-5.25) | 18.77 (17.63-19.91) | <0.001 | 1.31 (1.18-1.43) |
| EQ-5D-5L+SL | 5.61 (5.02-6.20) | 20.63 (19.47-21.79) | <0.001 | 1.37 (1.24-1.50) |
| EQ-5D-5L+SL+CG | 5.61 (5.03-6.19) | 20.54 (19.4-21.67) | <0.001 | 1.39 (1.26-1.52) |
| **Stroke** |  |  |  |  |
| **CN (n=70)** |  |  |  |  |
| EQ-5D-5L | 2.69 (2.39-3.00) | 20.47 (18.87-22.06) | <0.001 | 2.71 (2.44-2.97) |
| EQ-5D-5L+BR | 2.43 (2.14-2.71) | 19.72 (18.2-21.24) | <0.001 | 2.80 (2.54-3.07) |
| EQ-5D-5L+BR+SL | 3.09 (2.79-3.38) | 21.28 (19.71-22.85) | <0.001 | 2.86 (2.59-3.12) |
| **NL (n=109)** |  |  |  |  |
| EQ-5D-5L | 4.66 (4.13-5.19) | 25.28 (23.32-27.24) | <0.001 | 2.11 (1.89-2.33) |
| EQ-5D-5L+CG | 4.81 (4.3-5.31) | 26.30 (24.42-28.17) | <0.001 | 2.30 (2.08-2.52) |
| EQ-5D-5L+CG+BR | 4.43 (3.95-4.91) | 25.66 (23.88-27.44) | <0.001 | 2.39 (2.16-2.62) |
| **Anxiety** |  |  |  |  |
| **CN (n=391)** |  |  |  |  |
| EQ-5D-5L | 2.72 (2.41-3.04) | 13.30 (12.61-14.00) | <0.001 | 1.54 (1.42-1.66) |
| EQ-5D-5L+SL | 3.45 (3.13-3.78) | 15.64 (14.93-16.36) | <0.001 | 1.74 (1.61-1.86) |
| EQ-5D-5L+SL+TI | 4.28 (3.94-4.63) | 17.65 (16.90-18.41) | <0.001 | 1.80 (1.68-1.93) |
| EQ-5D-5L+SL+TI+CG | 4.56 (4.21-4.91) | 18.20 (17.50-19.00) | <0.001 | 1.81 (1.69-1.94) |
| **NL (n=435)** |  |  |  |  |
| EQ-5D-5L | 4.68 (4.06-5.29) | 24.72 (23.62-25.83) | <0.001 | 1.75 (1.63-1.88) |
| EQ-5D-5L+CO | 5.58 (4.96-6.20) | 27.52 (26.41-28.63) | <0.001 | 1.91 (1.78-2.04) |
| EQ-5D-5L+CO+SR | 5.66 (5.05-6.28) | 28.07 (26.96-29.18) | <0.001 | 1.96 (1.83-2.09) |
| EQ-5D-5L+CO+SR+TI | 7.19 (6.53-7.85) | 31.50 (30.40-32.70) | <0.001 | 1.99 (1.86-2.12) |
| EQ-5D-5L+CO+SR+TI+CG | 7.69 (6.98-8.40) | 34.00 (32.70-35.20) | <0.001 | 2.00 (1.87-2.13) |
| **Depression** |  |  |  |  |
| **CN (n=177)** |  |  |  |  |
| EQ-5D-5L | 2.68 (2.38-2.98) | 13.75 (12.76-14.74) | <0.001 | 1.73 (1.56-1.90) |
| EQ-5D-5L+SL | 3.41 (3.10-3.72) | 16.22 (15.19-17.25) | <0.001 | 1.92 (1.75-2.09) |
| EQ-5D-5L+SL+SR | 3.71 (3.39-4.02) | 17.13 (16.09-18.18) | <0.001 | 1.98 (1.81-2.15) |
| EQ-5D-5L+SL+SR+CG | 4.05 (3.72-4.38) | 18.00 (16.90-19.10) | <0.001 | 1.99 (1.81-2.16) |
| **NL (n=674)** |  |  |  |  |
| EQ-5D-5L | 4.67 (4.01-5.33) | 23.30 (22.35-24.25) | <0.001 | 1.52 (1.41-1.63) |
| EQ-5D-5L+CO | 5.55 (4.89-6.20) | 25.78 (24.84-26.73) | <0.001 | 1.66 (1.55-1.77) |
| EQ-5D-5L+CO+TI | 7.30 (6.59-8.01) | 29.79 (28.77-30.81) | <0.001 | 1.71 (1.60-1.82) |
| EQ-5D-5L+CO+TI+SR | 7.13 (6.43-7.83) | 29.70 (28.70-30.70) | <0.001 | 1.74 (1.63-1.85) |
| EQ-5D-5L+CO+TI+SR+SL | 7.70 (6.98-8.43) | 31.20 (30.20-32.20) | <0.001 | 1.76 (1.65-1.87) |
| **Skin disease** |  |  |  |  |
| **CN (n=342)** |  |  |  |  |
| EQ-5D-5L | 2.69 (2.38-2.99) | 9.58 (8.86-10.30) | <0.001 | 1.04 (0.92-1.16) |
| **NL (n=355)** |  |  |  |  |
| EQ-5D-5L | 4.66 (4.06-5.25) | 19.94 (18.75-21.14) | <0.001 | 1.39 (1.25-1.52) |
| EQ-5D-5L+CO | 5.45 (4.86-6.04) | 20.83 (19.65-22.01) | <0.001 | 1.41 (1.28-1.54) |
| EQ-5D-5L+CO+SL | 6.31 (5.68-6.93) | 22.70 (21.44-23.95) | <0.001 | 1.42 (1.29-1.55) |
| **Sleep disorder** |  |  |  |  |
| **CN (n=669)** |  |  |  |  |
| EQ-5D-5L | 2.85 (2.50-3.21) | 11.81 (11.20-12.41) | <0.001 | 1.17 (1.07-1.27) |
| EQ-5D-5L+SL | 3.57 (3.22-3.93) | 14.97 (14.37-15.58) | <0.001 | 1.48 (1.38-1.58) |
| EQ-5D-5L+SL+TI | 4.38 (4.01-4.76) | 16.62 (15.98-17.25) | <0.001 | 1.52 (1.42-1.62) |
| **NL (n=407)** |  |  |  |  |
| EQ-5D-5L | 4.68 (4.06-5.29) | 25.61 (24.46-26.77) | <0.001 | 1.83 (1.70-1.96) |
| EQ-5D-5L+SL | 5.59 (4.98-6.21) | 30.28 (29.13-31.43) | <0.001 | 2.17 (2.04-2.31) |
| EQ-5D-5L+SL+TI | 7.03 (6.38-7.67) | 33.16 (31.95-34.36) | <0.001 | 2.19 (2.06-2.33) |
| **Thyroid disease** |  |  |  |  |
| **CN (n=102)** |  |  |  |  |
| EQ-5D-5L | 2.68 (2.41-2.95) | 7.71 (6.56-8.87) | <0.001 | 0.87 (0.66-1.07) |
| **NL (n=194)** |  |  |  |  |
| EQ-5D-5L | 4.65 (4.11-5.19) | 18.99 (17.53-20.46) | <0.001 | 1.44 (1.27-1.6) |
| EQ-5D-5L+CG | 4.79 (4.26-5.31) | 19.52 (18.1-20.94) | <0.001 | 1.53 (1.36-1.70) |
| **Eating disorders** |  |  |  |  |
| **CN (n=80)** |  |  |  |  |
| EQ-5D-5L | 2.69 (2.39-2.98) | 15.08 (13.63-16.53) | <0.001 | 1.94 (1.70-2.17) |
| EQ-5D-5L+SL | 3.42 (3.11-3.72) | 16.99 (15.5-18.48) | <0.001 | 2.07 (1.83-2.31) |
| **NL (n=110)** |  |  |  |  |
| EQ-5D-5L | 4.68 (4.16-5.2) | 26.29 (24.45-28.13) | <0.001 | 2.23 (2.02-2.45) |
| EQ-5D-5L+CO | 5.59 (5.06-6.11) | 29.71 (27.86-31.56) | <0.001 | 2.48 (2.27-2.70) |
| EQ-5D-5L+CO+SR | 5.67 (5.14-6.2) | 30.53 (28.68-32.38) | <0.001 | 2.56 (2.34-2.78) |
| **Other mental disorders** |  |  |  |  |
| **CN (n=36)** |  |  |  |  |
| EQ-5D-5L | 2.67 (2.4-2.94) | 13.95 (11.94-15.97) | <0.001 | 1.94 (1.59-2.30) |
| EQ-5D-5L+SL | 3.41 (3.12-3.69) | 16.37 (14.26-18.49) | <0.001 | 2.13 (1.77-2.48) |
| EQ-5D-5L+SL+SR | 3.70 (3.41-3.99) | 17.43 (15.26-19.61) | <0.001 | 2.19 (1.83-2.55) |
| EQ-5D-5L+SL+SR+CO | 4.05 (3.73-4.36) | 18.90 (16.50-21.30) | <0.001 | 2.20 (1.84-2.56) |
| **NL (n=308)** |  |  |  |  |
| EQ-5D-5L | 4.67 (4.08-5.26) | 25.69 (24.45-26.94) | <0.001 | 1.92 (1.78-2.06) |
| EQ-5D-5L+CO | 5.57 (4.98-6.16) | 28.18 (26.94-29.42) | <0.001 | 2.08 (1.93-2.22) |
| EQ-5D-5L+CO+SR | 5.65 (5.07-6.24) | 28.98 (27.75-30.22) | <0.001 | 2.15 (2.01-2.30) |
| EQ-5D-5L+CO+SR+SL | 6.44 (5.83-7.05) | 31.10 (29.80-32.40) | <0.001 | 2.19 (2.04-2.33) |
| EQ-5D-5L+CO+SR+SL+CG | 6.97 (6.31-7.63) | 33.70 (32.30-35.10) | <0.001 | 2.21 (2.06-2.36) |
| **Obesity** |  |  |  |  |
| **CN (n=165)** |  |  |  |  |
| EQ-5D-5L | 2.68 (2.38-2.97) | 9.30 (8.32-10.28) | <0.001 | 1.04 (0.88-1.21) |
| **NL (n=364)** |  |  |  |  |
| EQ-5D-5L | 4.67 (4.04-5.3) | 23.77 (22.53-25.02) | <0.001 | 1.63 (1.50-1.76) |
| EQ-5D-5L+TI | 6.52 (5.86-7.18) | 26.91 (25.60-28.22) | <0.001 | 1.66 (1.53-1.79) |
| EQ-5D-5L+TI+BR | 5.89 (5.29-6.50) | 24.81 (23.61-26.02) | <0.001 | 1.68 (1.55-1.81) |
| EQ-5D-5L+TI+BR+SR | 5.88 (5.29-6.48) | 24.60 (23.40-25.70) | <0.001 | 1.69 (1.56-1.83) |

BR: breathing; CG: cognition; CN: China; CO: self-confidence; NL: Netherlands; SL: sleep; SR: social relationships; TI: tiredness

Known-groups validity was assessed using linear regressions. All known-groups comparisons were adjusted for age and sex.

Cohen's d values are reported in absolute terms. Effect sizes were interpreted as follows: 0.00–0.19 trivial, 0.20–0.49 small, 0.50–0.79 moderate, and ≥0.80 large.

Level sum scores (LSS) were calculated by summing the severity levels across the items of interest. To facilitate interpretation and comparability, LSS values were linearly rescaled to a 0-100 scale, where 0 indicates the best possible health state and 100 the worst.

**References**

1. Hays, R. D., Bjorner, J. B., Revicki, D. A., Spritzer, K. L., & Cella, D. (2009). Development of physical and mental health summary scores from the patient-reported outcomes measurement information system (PROMIS) global items. *Qual Life Res*, *18*(7), 873-880. https://doi.org/10.1007/s11136-009-9496-9
2. Yu, L., Buysse, D. J., Germain, A., Moul, D. E., Stover, A., Dodds, N. E., Johnston, K. L., & Pilkonis, P. A. (2011). Development of short forms from the PROMIS™ sleep disturbance and Sleep-Related Impairment item banks. *Behav Sleep Med*, *10*(1), 6-24. https://doi.org/10.1080/15402002.2012.636266
3. Al-Janabi, H., Flynn, T. N., & Coast, J. (2012). Development of a self-report measure of capability wellbeing for adults: the ICECAP-A. *Qual Life Res*, *21*(1), 167-176. https://doi.org/10.1007/s11136-011-9927-2
4. Topp, C. W., Østergaard, S. D., Søndergaard, S., & Bech, P. (2015). The WHO-5 Well-Being Index: a systematic review of the literature. *Psychother Psychosom*, *84*(3), 167-176. https://doi.org/10.1159/000376585
5. Bowling, A., Hankins, M., Windle, G., Bilotta, C., & Grant, R. (2013). A short measure of quality of life in older age: the performance of the brief Older People's Quality of Life questionnaire (OPQOL-brief). *Arch Gerontol Geriatr*, *56*(1), 181-187. https://doi.org/10.1016/j.archger.2012.08.012
6. Johnson, J. A., Janssen, M. F., Al Sayah, F., Bailey, H., Gandhi, M., Golicki, D., Gutacker, N., Lubetkin, E., Mulhern, B., Purba, F. D., Ramos-Goñi, J. M., Scott, D., Short, H., Sullivan, T., Viney, R., Yang, Z., & Zárate, V. (2025). EuroQol data for assessment of population health needs and instrument evaluation (EQ-DAPHNIE): a study for enhancing population health assessment. *Qual Life Res*. https://doi.org/10.1007/s11136-025-03983-2
7. Kroenke, K., Spitzer, R. L., & Williams, J. B. (2003). The Patient Health Questionnaire-2: validity of a two-item depression screener. *Med Care*, *41*(11), 1284-1292. https://doi.org/10.1097/01.Mlr.0000093487.78664.3c
8. Kroenke, K., Spitzer, R. L., Williams, J. B., Monahan, P. O., & Löwe, B. (2007). Anxiety disorders in primary care: prevalence, impairment, comorbidity, and detection. *Ann Intern Med*, *146*(5), 317-325. https://doi.org/10.7326/0003-4819-146-5-200703060-00004
9. Hoven, H., Eikemo, T. A., Backhaus-Hoven, I., Riebler, A., Fitzgerald, R., Martino, S., Huijts, T., Heggebø, K., Vidaurre-Teixidó, P., Bambra, C., & Balaj, M. (2025). The second Health Inequalities Module in the European Social Survey (ESS): Methodology and research opportunities. *Soc Sci Med*, *380*, 118228. https://doi.org/10.1016/j.socscimed.2025.118228
10. Rencz, F., & Janssen, M. F. (2024). Testing the Psychometric Properties of 9 Bolt-Ons for the EQ-5D-5L in a General Population Sample. *Value Health*, *27*(7), 943-954. https://doi.org/10.1016/j.jval.2024.03.2195
11. Choudhary, S. S., & Choudhary, S. R. (2009). Sleep effects on breathing and respiratory diseases. *Lung India*, *26*(4), 117-122. https://doi.org/10.4103/0970-2113.56345
12. Dignani, L., Toccaceli, A., Lucertini, C., Petrucci, C., & Lancia, L. (2016). Sleep and Quality of Life in People With COPD: A Descriptive-Correlational Study. *Clin Nurs Res*, *25*(4), 432-447. https://doi.org/10.1177/1054773815588515
13. Akbaş, E., & Filikci, S. B. (2025). The relationship between fatigue levels and psychosocial adjustment in elderly individuals with chronic obstructive pulmonary disease: A descriptive study. *Ir J Med Sci*, *194*(2), 649-656. https://doi.org/10.1007/s11845-025-03882-y
14. Sipowicz, K., Podlecka, M., Mokros, Ł., Pietras, T., & Łuczyńska, K. (2023). The feeling of loneliness and the sense of meaning in life in patients with various levels of bronchial asthma control. *J Asthma*, *60*(7), 1402-1408. https://doi.org/10.1080/02770903.2022.2151465
15. Battisha, A., Kahlon, A., & Kalra, D. K. (2025). Sleep-Disordered Breathing and Hypertension-A Systematic Review. *J Clin Med*, *14*(9). https://doi.org/10.3390/jcm14093115
16. Wang, Y., Chen, X. X., Lu, F. Y., Yan, Y. R., Li, S. Q., Zhang, L., Lin, Y. N., & Li, Q. Y. (2025). Association between sleep duration and hypertension risk in patients with obstructive sleep apnea. *NPJ Prim Care Respir Med*, *35*(1), 26. https://doi.org/10.1038/s41533-025-00429-7
17. Korhonen, P. E., Kivelä, S. L., Kautiainen, H., Järvenpää, S., & Kantola, I. (2011). Health-related quality of life and awareness of hypertension. *J Hypertens*, *29*(11), 2070-2074. https://doi.org/10.1097/HJH.0b013e32834bbca7
18. Banegas, J. R., López-García, E., Graciani, A., Guallar-Castillón, P., Gutierrez-Fisac, J. L., Alonso, J., & Rodríguez-Artalejo, F. (2007). Relationship between obesity, hypertension and diabetes, and health-related quality of life among the elderly. *Eur J Cardiovasc Prev Rehabil*, *14*(3), 456-462. https://doi.org/10.1097/HJR.0b013e3280803f29
19. Trevisol, D. J., Moreira, L. B., Kerkhoff, A., Fuchs, S. C., & Fuchs, F. D. (2011). Health-related quality of life and hypertension: a systematic review and meta-analysis of observational studies. *J Hypertens*, *29*(2), 179-188. https://doi.org/10.1097/HJH.0b013e328340d76f
20. Marwaha, K. (2022). Examining the Role of Psychosocial Stressors in Hypertension. *J Prev Med Public Health*, *55*(6), 499-505. https://doi.org/10.3961/jpmph.21.266
21. Bubulac, L., Zivari, M., Eremia, I. A., Erena, C., Gheorghe, C. M., Gheorghe, I. R., Tudor, V., Bogdan-Andreescu, C. F., Cadar, E., & Albu, C. C. (2025). Stress, Anxiety, and Self-Efficacy in Hypertension: Evidence from a Romanian Case-Control Study. *Diseases*, *13*(11). https://doi.org/10.3390/diseases13110373
22. Wang, L., Pan, H., Cai, Z., Li, X., & Luo, L. (2025). Association of social isolation, loneliness and risk of cardiovascular diseases: Meta-analysis of cohort studies. *BMC Public Health*, *25*(1), 3082. https://doi.org/10.1186/s12889-025-24300-z
23. Singh, M., Nag, A., Gupta, L., Thomas, J., Ravichandran, R., & Panjiyar, B. K. (2023). Impact of Social Support on Cardiovascular Risk Prediction Models: A Systematic Review. *Cureus*, *15*(9), e45836. https://doi.org/10.7759/cureus.45836
24. Boytsov, S., & Samorodskaya, I. (2023). Cardiovascular Diseases and Cognitive Impairments. *Neuroscience and Behavioral Physiology*, *53*, 186-192. https://doi.org/10.1007/s11055-023-01407-7
25. Gao, S., & Zhao, Y. (2023). Quality of life in postmenopausal women with osteoporosis: a systematic review and meta-analysis. *Qual Life Res*, *32*(6), 1551-1565. https://doi.org/10.1007/s11136-022-03281-1
26. Hu, J., Zheng, W., Zhao, D., Sun, L., Zhou, B., Liu, J., Wang, O., Jiang, Y., Xia, W., Xing, X., & Li, M. (2021). Health-related quality of life in men with osteoporosis: a systematic review and meta-analysis. *Endocrine*, *74*(2), 270-280. https://doi.org/10.1007/s12020-021-02792-0
27. Zhou, J., Hu, X., Zhou, S., Liu, T., & Chen, Z. (2026). Social isolation, loneliness, genetic susceptibility, and the hazard of incident osteoporosis. *Int J Surg*, *112*(1), 913-921. https://doi.org/10.1097/js9.0000000000003467
28. Lamichhane, A. P. (2005). Osteoporosis-an update. *JNMA J Nepal Med Assoc*, *44*(158), 60-66.
29. Kour, V., Swain, J., Singh, J., Singh, H., & Kour, H. (2024). A Review on Diabetic Retinopathy. *Curr Diabetes Rev*, *20*(6), e201023222418. https://doi.org/10.2174/0115733998253672231011161400
30. Vojtková, J., Ciljaková, M., Michnová, Z., & Turčan, T. (2012). Chronic complications of diabetes mellitus related to the respiratory system. *Pediatr Endocrinol Diabetes Metab*, *18*(3), 112-115.
31. Alanazi, A. H., Selim, M. S., Yendamuri, M. R., Zhang, D., Narayanan, S. P., & Somanath, P. R. (2025). The impact of diabetes mellitus on blood-tissue barrier regulation and vascular complications: Is the lung different from other organs? *Tissue Barriers*, *13*(2), 2386183. https://doi.org/10.1080/21688370.2024.2386183
32. Khandelwal, D., Dutta, D., Chittawar, S., & Kalra, S. (2017). Sleep Disorders in Type 2 Diabetes. *Indian J Endocrinol Metab*, *21*(5), 758-761. https://doi.org/10.4103/ijem.IJEM_156_17
33. Zhang, X., Su, Y., & Zhu, X. (2024). The association of diabetes with progression of sleep-disordered breathing based on a prospective cohort. *Diabetes Obes Metab*, *26*(9), 3935-3939. https://doi.org/10.1111/dom.15742
34. Kalra, S., & Sahay, R. (2018). Diabetes Fatigue Syndrome. *Diabetes Ther*, *9*(4), 1421-1429. https://doi.org/10.1007/s13300-018-0453-x
35. Fritschi, C., & Quinn, L. (2010). Fatigue in patients with diabetes: a review. *J Psychosom Res*, *69*(1), 33-41. https://doi.org/10.1016/j.jpsychores.2010.01.021
36. Kalra, S., Jena, B. N., & Yeravdekar, R. (2018). Emotional and Psychological Needs of People with Diabetes. *Indian J Endocrinol Metab*, *22*(5), 696-704. https://doi.org/10.4103/ijem.IJEM_579_17
37. Schwartz, M. D. (2002). Dyspepsia, peptic ulcer disease, and esophageal reflux disease. *West J Med*, *176*(2), 98-103.
38. Khanijow, V., Prakash, P., Emsellem, H. A., Borum, M. L., & Doman, D. B. (2015). Sleep Dysfunction and Gastrointestinal Diseases. *Gastroenterol Hepatol (N Y)*, *11*(12), 817-825.
39. Tuerk, E., Doss, S., & Polsley, K. (2023). Peptic Ulcer Disease. *Prim Care*, *50*(3), 351-362. https://doi.org/10.1016/j.pop.2023.03.003
40. Sonnenberg, A., & Everhart, J. E. (1997). Health impact of peptic ulcer in the United States. *Am J Gastroenterol*, *92*(4), 614-620.
41. Yegen, B. C. (2018). Lifestyle and Peptic Ulcer Disease. *Curr Pharm Des*, *24*(18), 2034-2040. https://doi.org/10.2174/1381612824666180510092303
42. Wen, Z., Li, X., Lu, Q., Brunson, J., Zhao, M., Tan, J., Wan, C., & Lei, P. (2014). Health related quality of life in patients with chronic gastritis and peptic ulcer and factors with impact: a longitudinal study. *BMC Gastroenterol*, *14*, 149. https://doi.org/10.1186/1471-230x-14-149
43. Ahmed, Z., Habib Ur Rehman, M., Abdul Rehman, F., Arshad, U., Zeeshan, M., Khan, K., Jabeen, M., & Khar, A. (2025). Association Between Gastroesophageal Reflux Disease and Chronic Respiratory Symptoms: A Systematic Review of Recent Clinical Evidence and Therapeutic Implications. *Cureus*, *17*(8), e91372. https://doi.org/10.7759/cureus.91372
44. Harding, S. M., Allen, J. E., Blumin, J. H., Warner, E. A., Pellegrini, C. A., & Chan, W. W. (2013). Respiratory manifestations of gastroesophageal reflux disease. *Ann N Y Acad Sci*, *1300*, 43-52. https://doi.org/10.1111/nyas.12231
45. Sajadinejad, M. S., Asgari, K., Molavi, H., Kalantari, M., & Adibi, P. (2012). Psychological issues in inflammatory bowel disease: an overview. *Gastroenterol Res Pract*, *2012*, 106502. https://doi.org/10.1155/2012/106502
46. Lackner, J. M., Gudleski, G. D., Dimuro, J., Keefer, L., & Brenner, D. M. (2013). Psychosocial predictors of self-reported fatigue in patients with moderate to severe irritable bowel syndrome. *Behav Res Ther*, *51*(6), 323-331. https://doi.org/10.1016/j.brat.2013.03.001
47. El-Salhy, M., Johansson, M., Klevstul, M., & Hatlebakk, J. G. (2025). Quality of life, functional impairment and healthcare experiences of patients with irritable bowel syndrome in Norway: an online survey. *BMC Gastroenterol*, *25*(1), 143. https://doi.org/10.1186/s12876-025-03685-6
48. Saeedinia, E., Poursharifi, H., Momeni, F., Vahedi, M., Sadeghi, A., Abdi, M., & Ghahremani, R. (2025). Psychological determinants of irritable bowel syndrome and its impact on quality of life: a machine learning approaches. *Gastroenterol Hepatol Bed Bench*, *18*(1), 100-114. https://doi.org/10.22037/ghfbb.v18i1.3082
49. Yazar, A., Atis, S., Konca, K., Pata, C., Akbay, E., Calikoglu, M., & Hafta, A. (2001). Respiratory symptoms and pulmonary functional changes in patients with irritable bowel syndrome. *Am J Gastroenterol*, *96*(5), 1511-1516. https://doi.org/10.1111/j.1572-0241.2001.03748.x
50. Jarrett, M., Heitkemper, M., Cain, K. C., Burr, R. L., & Hertig, V. (2000). Sleep disturbance influences gastrointestinal symptoms in women with irritable bowel syndrome. *Dig Dis Sci*, *45*(5), 952-959. https://doi.org/10.1023/a:1005581226265
51. Frändemark, Å., Jakobsson Ung, E., Törnblom, H., Simrén, M., & Jakobsson, S. (2017). Fatigue: a distressing symptom for patients with irritable bowel syndrome. *Neurogastroenterol Motil*, *29*(1). https://doi.org/10.1111/nmo.12898
52. Norlin, A. K., Walter, S., Icenhour, A., Keita Å, V., Elsenbruch, S., Bednarska, O., Jones, M. P., Simon, R., & Engström, M. (2021). Fatigue in irritable bowel syndrome is associated with plasma levels of TNF-α and mesocorticolimbic connectivity. *Brain Behav Immun*, *92*, 211-222. https://doi.org/10.1016/j.bbi.2020.11.035
53. Bengtsson, M., Sjöberg, K., Candamio, M., Lerman, A., & Ohlsson, B. (2013). Anxiety in close relationships is higher and self-esteem lower in patients with irritable bowel syndrome compared to patients with inflammatory bowel disease. *Eur J Intern Med*, *24*(3), 266-272. https://doi.org/10.1016/j.ejim.2012.11.011
54. Waliszewska-Prosół, M., Nowakowska-Kotas, M., Chojdak-Łukasiewicz, J., & Budrewicz, S. (2021). Migraine and Sleep-An Unexplained Association? *Int J Mol Sci*, *22*(11). https://doi.org/10.3390/ijms22115539
55. Almansour, N. A., Alsalamah, S. S., Alsubaie, R. S., Alshathri, N. N., Alhedyan, Y. A., & Althekair's, F. Y. (2025). Association between migraine severity and sleep quality: a nationwide cross-sectional study. *Front Neurol*, *16*, 1529213. https://doi.org/10.3389/fneur.2025.1529213
56. Awaki, E., Takeshima, T., Matsumori, Y., Hirata, K., Miyazaki, N., Takemura, R., Osaga, S., Tanizawa, Y., & Komori, M. (2024). Impact of Migraine on Daily Life: Results of the Observational survey of the Epidemiology, Treatment, and Care of Migraine (OVERCOME [Japan]) Study. *Neurol Ther*, *13*(1), 165-182. https://doi.org/10.1007/s40120-023-00569-3
57. Raggi, A., Giovannetti, A. M., Quintas, R., D'Amico, D., Cieza, A., Sabariego, C., Bickenbach, J. E., & Leonardi, M. (2012). A systematic review of the psychosocial difficulties relevant to patients with migraine. *J Headache Pain*, *13*(8), 595-606. https://doi.org/10.1007/s10194-012-0482-1
58. Abumilha, A. K. Y., Abukaftah, A. S. A., Al-Mudhi, M. M., Al Fareh, N. A., & Muflih Abudasser, A. (2025). Association between migraine and depression, anxiety, and stress in the Aseer region: a cross-sectional study. *Front Neurol*, *16*, 1650891. https://doi.org/10.3389/fneur.2025.1650891
59. Choudhary, A. K. (2024). Migraine and cognitive impairment: the interconnected processes. *Brain-Apparatus Communication: A Journal of Bacomics*, *3*(1), 2439437. https://doi.org/10.1080/27706710.2024.2439437
60. Gu, L., Wang, Y., & Shu, H. (2022). Association between migraine and cognitive impairment. *J Headache Pain*, *23*(1), 88. https://doi.org/10.1186/s10194-022-01462-4
61. Cramer, S. C., Richards, L. G., Bernhardt, J., & Duncan, P. (2023). Cognitive Deficits After Stroke. *Stroke*, *54*(1), 5-9. https://doi.org/10.1161/strokeaha.122.041775
62. Sand, K. M., Wilhelmsen, G., Naess, H., Midelfart, A., Thomassen, L., & Hoff, J. M. (2016). Vision problems in ischaemic stroke patients: effects on life quality and disability. *Eur J Neurol*, *23 Suppl 1*, 1-7. https://doi.org/10.1111/ene.12848
63. Barnett, H. M., Davis, A. P., & Khot, S. P. (2022). Stroke and breathing. *Handb Clin Neurol*, *189*, 201-222. https://doi.org/10.1016/b978-0-323-91532-8.00016-1
64. Lisabeth, L. D., Sánchez, B. N., Lim, D., Chervin, R. D., Case, E., Morgenstern, L. B., Tower, S., & Brown, D. L. (2019). Sleep-disordered breathing and poststroke outcomes. *Ann Neurol*, *86*(2), 241-250. https://doi.org/10.1002/ana.25515
65. Chen, W., Jiang, T., Huang, H., & Zeng, J. (2023). Post-stroke fatigue: a review of development, prevalence, predisposing factors, measurements, and treatments. *Front Neurol*, *14*, 1298915. https://doi.org/10.3389/fneur.2023.1298915
66. Simpson, D. B., Gay, C. L., Berg, S., English, C., Ihle-Hansen, H., Berg Helland, G., Larsson, P., Skogestad, I., Stubberud, J., Øverland, B., & Lerdal, A. (2026). Sleep quality, not sleep apnoea, is associated with post-stroke fatigue in acute ischemic stroke: a cross-sectional Norwegian study (NORFAST). *Disabil Rehabil*, 1-11. https://doi.org/10.1080/09638288.2026.2620503
67. Elendu, C., Amaechi, D. C., Elendu, T. C., Ibhiedu, J. O., Egbunu, E. O., Ndam, A. R., Ogala, F., Ologunde, T., Peterson, J. C., Boluwatife, A. I., Okongko, A. O., Fatoye, J. O., Akpovona, O. L., Onyekweli, S. O., Temitope, A. Y., Achimugu, A. O., & Temilade, A. V. (2023). Stroke and cognitive impairment: understanding the connection and managing symptoms. *Ann Med Surg (Lond)*, *85*(12), 6057-6066. https://doi.org/10.1097/ms9.0000000000001441
68. Thompson, H. S., & Ryan, A. (2009). The impact of stroke consequences on spousal relationships from the perspective of the person with stroke. *J Clin Nurs*, *18*(12), 1803-1811. https://doi.org/10.1111/j.1365-2702.2008.02694.x
69. Horne, J., Lincoln, N. B., Preston, J., & Logan, P. (2014). What does confidence mean to people who have had a stroke? A qualitative interview study. *Clin Rehabil*, *28*(11), 1125-1135. https://doi.org/10.1177/0269215514534086
70. Chellappa, S. L., & Aeschbach, D. (2022). Sleep and anxiety: From mechanisms to interventions. *Sleep Med Rev*, *61*, 101583. https://doi.org/10.1016/j.smrv.2021.101583
71. Pollack, M. H., Endicott, J., Liebowitz, M., Russell, J., Detke, M., Spann, M., Ball, S., & Swindle, R. (2008). Examining quality of life in patients with generalized anxiety disorder: clinical relevance and response to duloxetine treatment. *J Psychiatr Res*, *42*(12), 1042-1049. https://doi.org/10.1016/j.jpsychires.2007.11.006
72. Kroenke, K., Strine, T. W., Spitzer, R. L., Williams, J. B., Berry, J. T., & Mokdad, A. H. (2009). The PHQ-8 as a measure of current depression in the general population. *J Affect Disord*, *114*(1-3), 163-173. https://doi.org/10.1016/j.jad.2008.06.026
73. Pandi-Perumal, S. R., Monti, J. M., Burman, D., Karthikeyan, R., BaHammam, A. S., Spence, D. W., Brown, G. M., & Narashimhan, M. (2020). Clarifying the role of sleep in depression: A narrative review. *Psychiatry Res*, *291*, 113239. https://doi.org/10.1016/j.psychres.2020.113239
74. Czerwińska, A., & Pawłowski, T. (2020). Cognitive dysfunctions in depression - significance, description and treatment prospects. *Psychiatr Pol*, *54*(3), 453-466. https://doi.org/10.12740/PP/OnlineFirst/105415 (Original work published Zaburzenia funkcji poznawczych w depresji – znaczenie, charakterystyka oraz możliwości leczenia.)
75. Cameron, S., Donnelly, A., Broderick, C., Arichi, T., Bartsch, U., Dazzan, P., Elberling, J., Godfrey, E., Gringras, P., Heathcote, L. C., Joseph, D., Wood, T. C., Pariante, C., Rubia, K., & Flohr, C. (2024). Mind and skin: Exploring the links between inflammation, sleep disturbance and neurocognitive function in patients with atopic dermatitis. *Allergy*, *79*(1), 26-36. https://doi.org/10.1111/all.15818
76. Skoie, I. M., Ternowitz, T., Jonsson, G., Norheim, K., & Omdal, R. (2015). Fatigue in psoriasis: a phenomenon to be explored. *Br J Dermatol*, *172*(5), 1196-1203. https://doi.org/10.1111/bjd.13647
77. Villalon-Gomez, J. M. (2018). Pityriasis Rosea: Diagnosis and Treatment. *Am Fam Physician*, *97*(1), 38-44.
78. Zeiser, K., Hammel, G., Kirchberger, I., & Traidl-Hoffmann, C. (2021). Social and psychosocial effects on atopic eczema symptom severity - a scoping review of observational studies published from 1989 to 2019. *J Eur Acad Dermatol Venereol*, *35*(4), 835-843. https://doi.org/10.1111/jdv.16950
79. Rousset, L., & Halioua, B. (2018). Stress and psoriasis. *Int J Dermatol*, *57*(10), 1165-1172. https://doi.org/10.1111/ijd.14032
80. Feldman, S. R., Malakouti, M., & Koo, J. Y. (2014). Social impact of the burden of psoriasis: effects on patients and practice. *Dermatol Online J*, *20*(8).
81. Słomian, A., Łakuta, P., Bergler-Czop, B., & Brzezińska-Wcisło, L. (2018). Self-esteem is related to anxiety in psoriasis patients: A case control study. *J Psychosom Res*, *114*, 45-49. https://doi.org/10.1016/j.jpsychores.2018.09.005
82. Kong, J., Zhou, L., Li, X., & Ren, Q. (2023). Sleep disorders affect cognitive function in adults: an overview of systematic reviews and meta-analyses. *Sleep Biol Rhythms*, *21*(2), 133-142. https://doi.org/10.1007/s41105-022-00439-9
83. Hyndych, A., El-Abassi, R., & Mader, E. C., Jr. (2025). The Role of Sleep and the Effects of Sleep Loss on Cognitive, Affective, and Behavioral Processes. *Cureus*, *17*(5), e84232. https://doi.org/10.7759/cureus.84232
84. Kent, R. G., Uchino, B. N., Cribbet, M. R., Bowen, K., & Smith, T. W. (2015). Social Relationships and Sleep Quality. *Ann Behav Med*, *49*(6), 912-917. https://doi.org/10.1007/s12160-015-9711-6
85. Lemola, S., Räikkönen, K., Gomez, V., & Allemand, M. (2013). Optimism and self-esteem are related to sleep. Results from a large community-based sample. *Int J Behav Med*, *20*(4), 567-571. https://doi.org/10.1007/s12529-012-9272-z
86. Kapan, A., Ristic, M., Felsinger, R., & Waldhoer, T. (2025). Association of Self-Perceived Fatigue, Muscle Fatigue, and Sleep Disorders with Cognitive Function in Older Adults: A Cross-Sectional Study. *J Am Med Dir Assoc*, *26*(4), 105477. https://doi.org/10.1016/j.jamda.2024.105477
87. Szelog, J., Swanson, H., Sniegowski, M. C., & Lyon, D. B. (2022). Thyroid Eye Disease. *Mo Med*, *119*(4), 343-350.
88. Nazem, M. R., Bastanhagh, E., Emami, A., Hedayati, M., Samimi, S., & Karami, M. (2021). The relationship between thyroid function tests and sleep quality: cross-sectional study. *Sleep Sci*, *14*(3), 196-200. https://doi.org/10.5935/1984-0063.20200050
89. Grixti, L., Fisher, H., Priestley, J., McMullan, C., Woollven, A., Perros, P., Mitchell, A. L., Gan, E. H., & Pearce, S. H. (2025). Prevalence and severity of fatigue in treated hypothyroidism: results of a UK survey. *Eur Thyroid J*, *14*(3). https://doi.org/10.1530/etj-25-0044
90. Nicola Marioara, O. M., Popescu, M., Vlădoianu, C. N., Carlig, V., Carsote, M., & Ghenea, A. E. (2021). Study of Cognitive Disfunctions in Thyroid Pathology. *Curr Health Sci J*, *47*(2), 256-262. https://doi.org/10.12865/chsj.47.02.16
91. Al Qaderi, A. H., Osman, A. A., Manasrah, H., Ali, M. Z., & Al Qaderi, N. (2026). Exploring the Connection Between Thyroid Health and Psychiatric Disorders: A Comprehensive Review With a Focus on Schizophrenia and Bipolar Disorder. *Cureus*, *18*(1), e102146. https://doi.org/10.7759/cureus.102146
92. Fan, J., Zhou, K., & Yu, C. (2025). Causal Relationship Between Emotional Disorders and Thyroid Disorders: A Bidirectional Two-Sample Mendelian Randomization Study. *Brain Behav*, *15*(1), e70252. https://doi.org/10.1002/brb3.70252
93. Martins-de-Passos, T. O., Mesas, A. E., Beneit, N., Díaz-Goñi, V., Peral-Martinez, F., Cekrezi, S., Martinez-Vizcaino, V., & Jimenez-Lopez, E. (2024). Are Sleep Parameters and Chronotype Associated with Eating Disorder Risk? A Cross-Sectional Study of University Students in Spain. *J Clin Med*, *13*(18). https://doi.org/10.3390/jcm13185482
94. Bazzani, A., & Faraguna, U. (2025). Eat Well, Sleep Well: Exploring the Association Between Eating Behavior and Sleep Quality. *Nutrients*, *17*(17). https://doi.org/10.3390/nu17172908
95. Meneguzzo, P., Marzotto, A., Conti, F., Mezzani, B., Maggi, L., & Todisco, P. (2025). Emotional intelligence and loneliness in eating disorders: a cluster-analytic study across diagnostic categories. *J Eat Disord*, *13*(1), 221. https://doi.org/10.1186/s40337-025-01411-x
96. Cortés-García, L., Rodríguez-Cano, R., & von Soest, T. (2022). Prospective associations between loneliness and disordered eating from early adolescence to adulthood. *Int J Eat Disord*, *55*(12), 1678-1689. https://doi.org/10.1002/eat.23793
97. Abdoli, M., Schiechtl, E., Rosato, M. S., Mangweth-Matzek, B., Cotrufo, P., & Hüfner, K. (2025). Body image, self-esteem, emotion regulation, and eating disorders in adults: a systematic review. *Neuropsychiatr*, *39*(3), 118-132. https://doi.org/10.1007/s40211-025-00544-4 (Original work published Körperbild, Selbstwert, Emotionsregulation und Essstörungen bei Erwachsenen: eine systematische Übersicht.)
98. Diaz-Marsa, M., Pemau, A., de la Torre-Luque, A., Vaz-Leal, F., Rojo-Moreno, L., Beato-Fernandez, L., Graell, M., Carrasco-Diaz, A., & Carrasco, J. L. (2023). Executive dysfunction in eating disorders: Relationship with clinical features. *Prog Neuropsychopharmacol Biol Psychiatry*, *120*, 110649. https://doi.org/10.1016/j.pnpbp.2022.110649
99. Weider, S., Indredavik, M. S., Lydersen, S., & Hestad, K. (2015). Neuropsychological function in patients with anorexia nervosa or bulimia nervosa. *Int J Eat Disord*, *48*(4), 397-405. https://doi.org/10.1002/eat.22283
100. Baglioni, C., Nanovska, S., Regen, W., Spiegelhalder, K., Feige, B., Nissen, C., Reynolds, C. F., & Riemann, D. (2016). Sleep and mental disorders: A meta-analysis of polysomnographic research. *Psychol Bull*, *142*(9), 969-990. https://doi.org/10.1037/bul0000053
101. Patin, A., & Hurlemann, R. (2015). Social cognition. *Handb Exp Pharmacol*, *228*, 271-303. https://doi.org/10.1007/978-3-319-16522-6_10
102. Dixon, A. E., & Peters, U. (2018). The effect of obesity on lung function. *Expert Rev Respir Med*, *12*(9), 755-767. https://doi.org/10.1080/17476348.2018.1506331
103. Bonsignore, M. R. (2022). Obesity and Obstructive Sleep Apnea. *Handb Exp Pharmacol*, *274*, 181-201. https://doi.org/10.1007/164_2021_558
104. Hajek, A., & König, H. H. (2019). Obesity and loneliness. Findings from a longitudinal population-based study in the second half of life in Germany. *Psychogeriatrics*, *19*(2), 135-140. https://doi.org/10.1111/psyg.12375
105. Stunkard, A. J., & Wadden, T. A. (1992). Psychological aspects of severe obesity. *Am J Clin Nutr*, *55*(2 Suppl), 524s-532s. https://doi.org/10.1093/ajcn/55.2.524s
